# Supplementary material for: Neoadjuvant modified FOLFIRINOX plus nivolumab in borderline-resectable pancreatic ductal adenocarcinoma: a pilot phase 1 trial
Source: Nat Commun. 2026 Jan 31;17:2232. doi: 10.1038/s41467-026-68976-2 (PMC12963377; doi:10.1038/s41467-026-68976-2)
Supplement: Supplementary file 1 — Supplementary Information [file 41467_2026_68976_MOESM1_ESM.pdf]

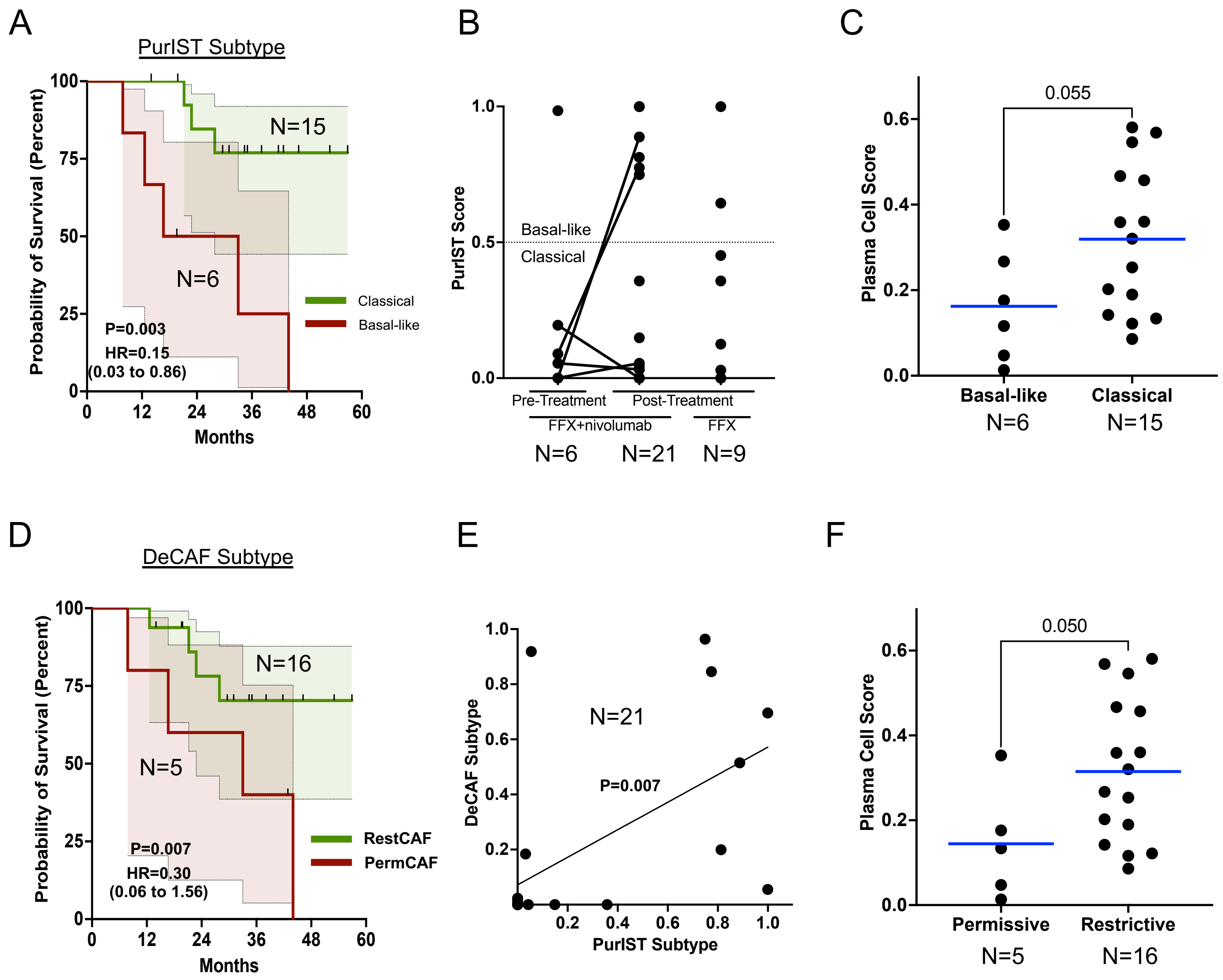

Supplementary Figure 1: A) Kaplan Meier plot of overall survival from trial patients distinguished by classical (PurIST score <0.5) or basal-like (PurIST score >0.5) subtype of post-treatment resected tumors. P value from Log rank Mantel Cox test and hazard ratio with 95% confidence intervals are given. B) PurIST scores for pre- and post-treatment trial tumors and tumors treated with FOLFIRINOX alone. C) CIBERSORT-ABS plasma cell scores from FFX and nivolumab treated resection specimens are shown for both classical and basal-like tumor subtypes. P values are from a tw-tailed Mann-Whitney test. D) Kaplan Meier plot of overall survival from trial patients distinguished by restrictive CAF subtype (DeCAF score <0.5) versus permissive CAF subtype (DeCAF score >0.5) post-treatment resected tumors. P value from Log rank Mantel Cox test and hazard ratio with 95% confidence intervals are given. E) Correlation between DeCAF and PurIST subtype scores for resected trial tumors. F) CIBERSORT-ABS scores for plasma cells from FFX and nivolumab treated resection specimens are shown for both permissive and restrictive CAF subtypes. P values are from a Mann-Whitney test.

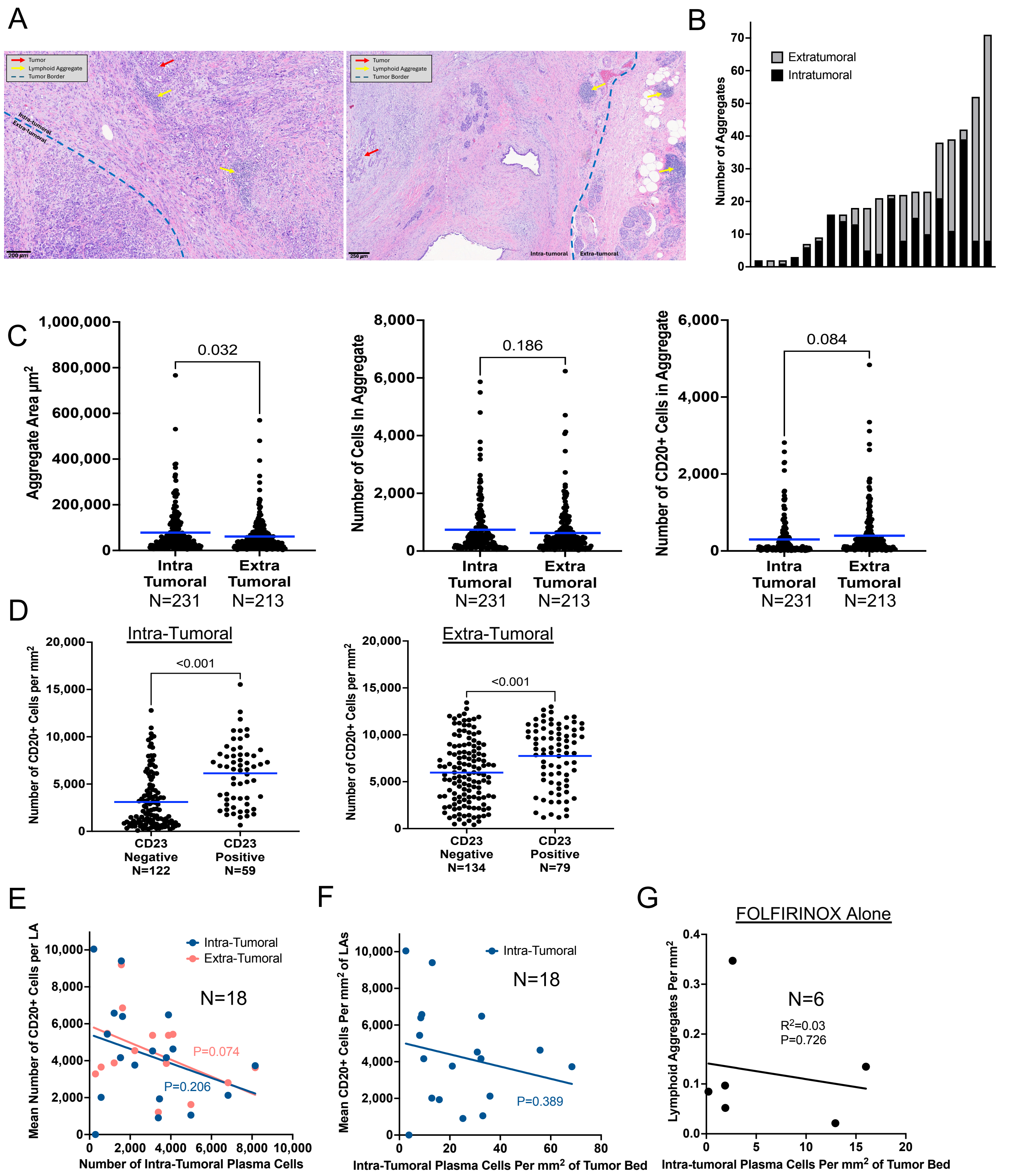

Supplementary Figure 2: A) The boundary demarcating the tumor bed (blue dashed line) used to define intra-tumoral and extra-tumoral LAs (yellow arrows). Red arrows indicate tumor cells. Scale bars represent 250 µm. B) The number of intra-tumoral (N=213) and extra-tumoral (N=233) LAs identified for each of the 20 trial patient tumors analyzed. C) Comparisons of intra-tumoral and extra-tumoral LAs by (from left to right) area, total cell number, and CD20+ cell number. D) The density of CD20+ cells correlates with CD23+ staining for both intra-tumoral (left panel) and extra-tumoral (right panel) LAs. E) The number of intra-tumoral plasma cells per tumor does not positively correlate with the mean number of CD20+ cells per LA (intra-tumoral or extra-tumoral). F) The intra-tumoral density of plasma cells does not correlate with the mean density of CD20+ cells per intra-tumoral LA. G) No apparent correlation between intra-tumoral LA density and plasma cell density for patients treated with FOLFIRINOX alone. For C and D, P values were derived from two-tailed t tests. For E-G, P values were derived from two-tailed Pearson correlation.

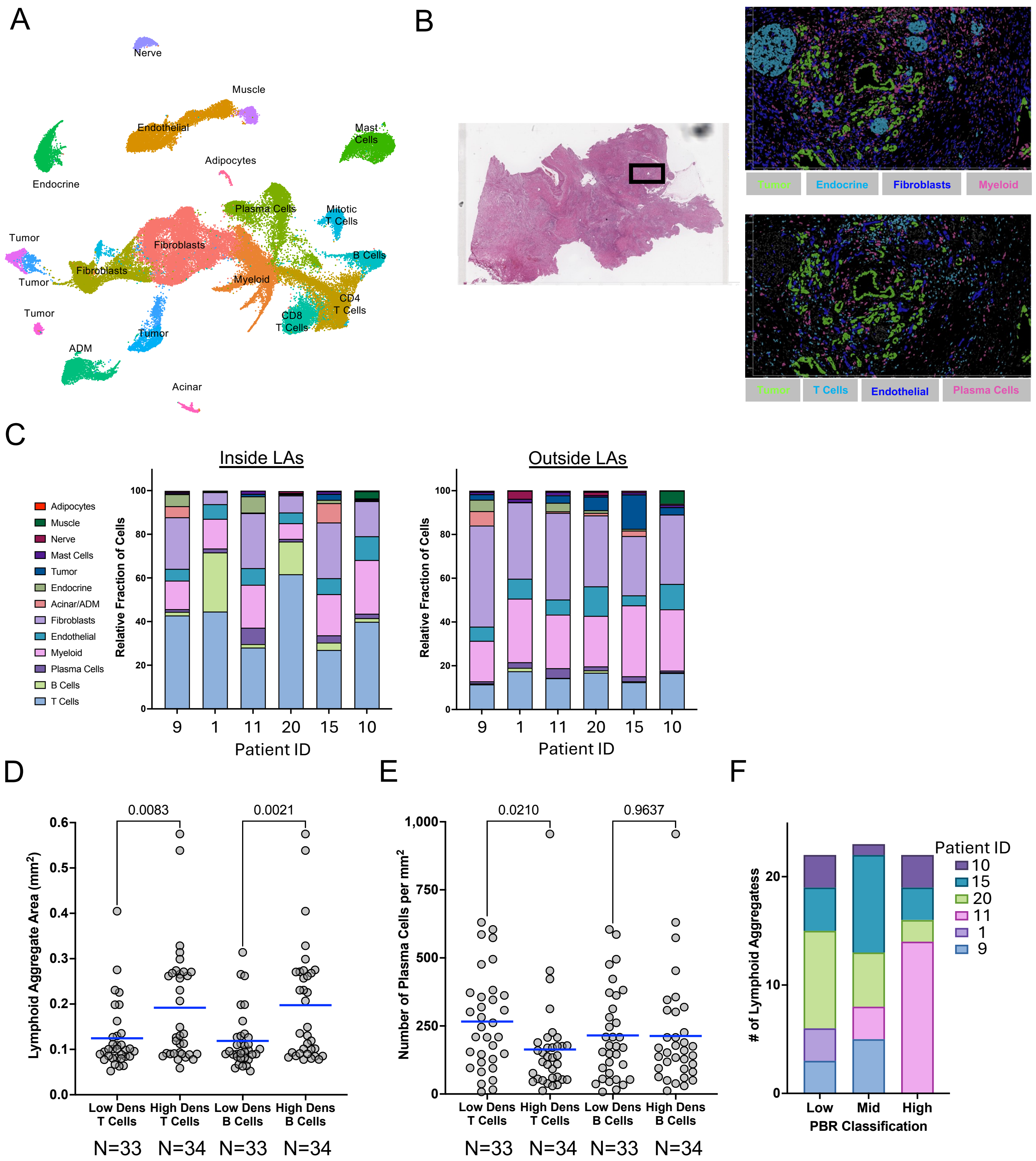

Supplementary Figure 3: A) UMAP of cell types identified from the Xenium 5K Prime Assay. Cluster size appearance on UMAP is not proportional to abundance of the cell type in the tissue. B) H&E-stained tissue section used for Xenium spatial transcriptomics (left image); Cell types from annotated UMAP clustering mapped back to the original image from spatial transcriptomics (middle and right images), black box on H&E image represents area shown in middle and right images. Distance between scale bar tics represents 100 μm. C) Relative fractions of each cell type identified inside (left) or outside (right) LAs for each of 6 patient samples. D) Area of LAs distinguished by high (top 50%) or low (bottom 50%) T and B cell densities. P values were derived from two-tailed t tests. E) Plasma cell density of LAs distinguished by high (top 50%) or low (bottom 50%) T and B cell densities. P values were derived from two-tailed t tests. F) The number of LAs for each patient classified by tertiles of PBR.

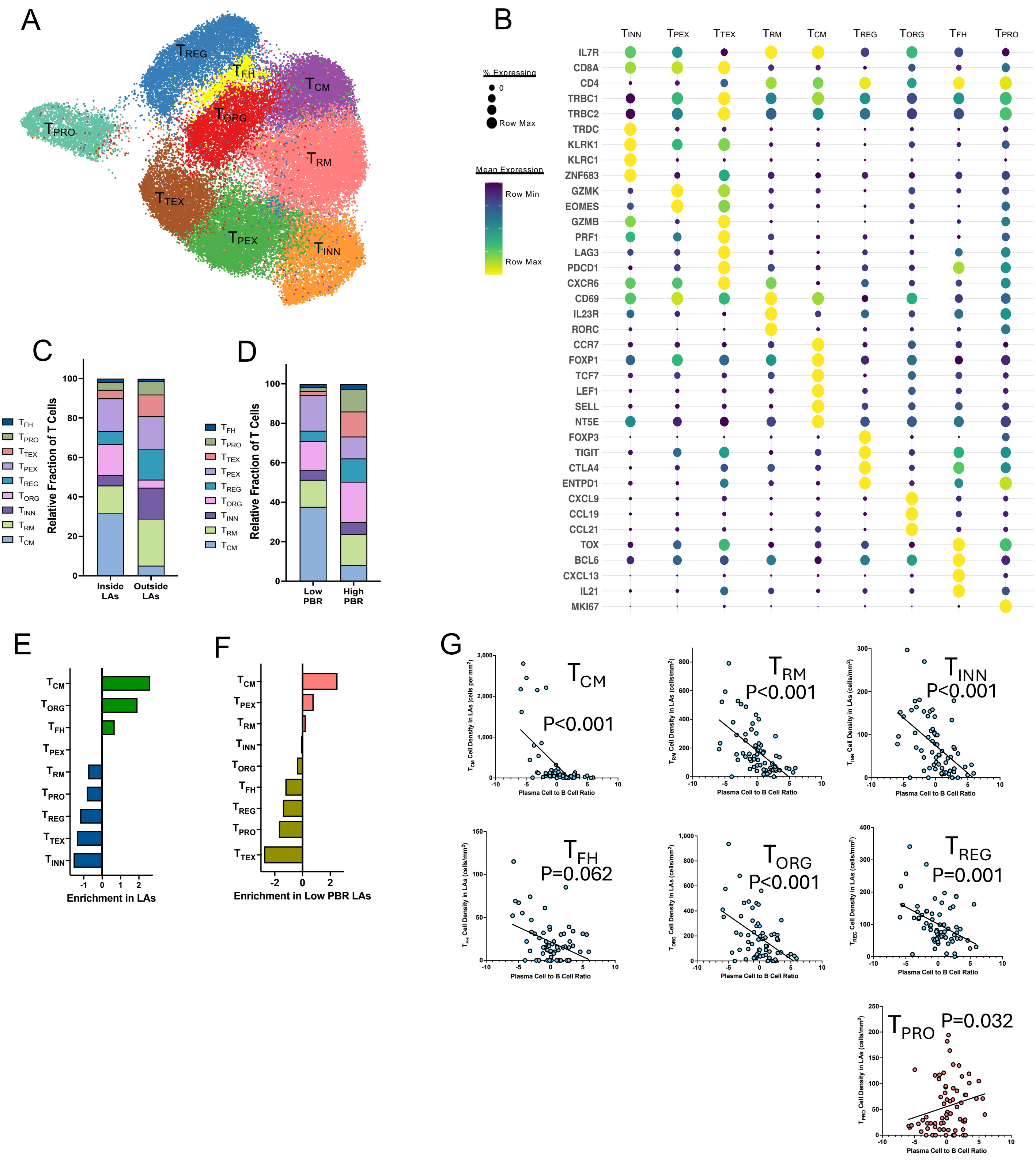

Supplementary Figure 4: A) Clustering of T cells from the Xenium 5K Prime assay. B) Phenotypic markers of T cell subsets; percentage of cells expressing each gene is represented by bubble size ranging from 0% to the maximum for each gene, the mean expression color scale ranges from minimum to maximum expression for each gene. C) The relative fractions of each T cell subset inside and outside the 67 LAs. D) The relative fractions of each T cell subset in low PBR and high PBR LAs. E) Relative enrichment of T cell subsets inside LAs. F) Relative enrichment of T cell subsets inside LAs with low PBR. G) Correlation of the PBR to densities of non-cytolytic T cell subsets. Trend lines plotted from simple linear regression, P values from Spearman non-parametric correlation. Data represent 67 LAs.

ANTIBODIES	SOURCE	Dilution	IDENTIFIER
Monoclonal mouse anti-human CD20cy	Dako	1:200	Catalog #: M0755; Clone: L26; Lot 00064779
Polyclonal rabbit anti-human CD3	Dako	1:100	Catalog #: A0452; Clone: Poly; Lot 41654191
Monoclonal mouse anti-human CD23	Biocare Medical	1:50	Catalog #: CM100A; Clone: 1B12; Lot 103123A
Monoclonal mouse anti-human MUM1	Dako	1:200	Catalog #: GA644; Clone: MUM1p; Lot 41608598
**REAGENTS**			
AR6 antigen retrieval buffer	Akoya Biosciences		Catalog #: AR600250ML
AR9 antigen retrieval buffer	Akoya Biosciences		Catalog #: AR900250ML
Bond primary antibody diluent	Leica		Catalog #: AR9352
EnVision+/HRP, Rabbit, HRP. Rabbit	Agilent		Catalog #: K4003
EnVision+/HRP, Mouse, HRP. Mouse	Agilent		Catalog #: K400111-2
BOND Polymer Refine Detection	Leica		Catalog #: DS9800

Supplementary Table 1 : Key Immunohistochemistry Resources

A Pilot and Feasibility Study of PD-1 Blockade with Nivolumab in combination with chemotherapy in patients with Borderline Resectable Pancreatic Adenocarcinoma

Protocol Number:

Study Drug: Nivolumab, FFX (5-FU, leucovorin, oxaliplatin, irinotecan)

Version Number: **4**.0

Version Date: 3/27/2019

IND Number:

<u>Principal Investigator (Sponsor-Investigator)</u>

Zev A. Wainberg
University of California Los Angeles
Jonsson Comprehensive Cancer Center, Los Angeles, CA 90095
Telephone: 310-829-5471
E-mail: zwainberg@mednet.ucla.edu

<u>Co-Investigators-University of California Los Angeles</u>

Timothy Donahue, MD
Joe Hines, MD
Lee Rosen, MD
Jonathan Goldman, MD, PhD
J. Randolph Hecht, MD

Revision History

Version 1.0	12JUNL2018
Version 2.0	14NOV2018
Version 3.0	26FEB2019
Version 4.0	27MAR2019

Protocol Signature Page
Protocol No: **Version Date:** 27MAR2019

1. I agree to follow this protocol version as approved by the UCLA Protocol Review Committee (PRC), Committee on Human Research (CHR), and Data Safety Monitoring Committee (DSMC).
2. I will conduct the study in accordance with applicable CHR requirements, Federal regulations, and state and local laws to maintain the protection of the rights and welfare of study participants.
3. I certify that I, and the study staff, have received the requisite training to conduct this research protocol.
4. I have read and understand the information in the Investigators' Brochure (or Manufacturer's Brochure) regarding the risks and potential benefits. I agree to conduct the protocol in accordance with Good Clinical Practices (ICH-GCP), the applicable ethical principles, the Statement of Investigator (Form FDA 1572), and with local regulatory requirements. In accordance with the FDA Modernization Act, I will ensure the registration of the trial on the www.clinicaltrials.gov website.
5. I agree to maintain adequate and accurate records in accordance with CHR policies, Federal, state and local laws and regulations.

UCLA Principal Investigator / Study Chair

____
Printed Name

____________________________________ ________________
Signature Date

Principal Investigator **Site**

____________________________________ ________________
Printed Name

____________________________________ ________________
Signature Date

PROTOCOL SYNOPSIS

**TITLE**	A Pilot and Feasibility Study of PD-1 Blockade with Nivolumab in combination with chemotherapy in patients with Borderline Resectable Pancreatic Adenocarcinoma
**FUNDING**	BMS<br><br>UCLA
**INVESTIGATORS**	Zev A. Wainberg, MD (PI)<br>Timothy Donahue, MD<br>Joe Hines, MD<br>Lee Rosen, MD<br>Jonathan Goldman, MD<br>J. Randolph Hecht, MD
**NUMBER OF SITES**	1 (UCLA Jonsson Comprehensive Cancer Center)
**RATIONALE**	Pancreatic ductal adenocarcinoma (PDAC) remains one of the most challenging cancers to treat due to its advanced stage at the time of diagnosis, its aggressive behavior and its resistance to treatment. While surgery provides the highest chance of cure for patients with localized PDAC, patients with either upfront resectable or borderline resectable disease may benefit from neoadjuvant therapy to select appropriate candidates for potentially curative operations, eradicate micrometastases, and increase the likelihood of R0 resection rate.<br><br>Recent evidence has emerged suggesting that PDAC is particularly non-immunogenic due in part to a complex tumor microenvironment consisting of multiple components including pancreatic stellate cells (PSC), endothelial cells, immune cells and fibroblasts with an extensive paracrine and autocrine network residing in a stiff extracellular matrix. The desmosplastic reaction that is characteristic of the PDAC stroma has been associated with increased cell proliferation and migration leading to metastasis in PDAC, while it disturbs the pancreatic tissue architecture by changing the layout of blood and lymphatic vessels.<br><br>The programmed cell death 1 (PD-1) pathway represents a major immune checkpoint, which may be hijacked by tumor cells to overcome active T-cell immune surveillance. Preclinical *in vitro* and *in vivo* experiments have shown that PD-1 blockade using Nivolumab enhances tumor-specific T-cell activation, T cell infiltration into the tumors, cytokine production, anti-tumor effector function, and clearance of tumor cells by the immune system. In

some malignancies, responding patients showed proliferation of intra-tumural CD8+ T-cells that directly correlated with radiographic reduction in tumor size. Pre-treatment samples obtained from responding patients showed higher numbers of CD8, PD1, and PD-L1 expressing cells at the invasive tumor margin and inside tumors. These findings indicate that tumor regression following therapeutic PD-1 blockade requires pre-existing CD8+ T cells that are negatively regulated by PD-1/PD-L1 mediated adaptive immune resistance.

Furthermore, pre-clinical studies with Nivolumab and chemotherapy have also shown that increased neo-antigen formation may prime certain tumors to respond to chemotherapy**.** It is currently unknown which antigens are being recognized by T cells attacking tumors after release of PD-1 blockade. But evidence from studies with other immunotherapies suggests that most of these antigens are neo-epitopes resulting from somatic mutations.

We selected FOLFIRINOX as the chemotherapy backbone due to its safety and efficacy profile reported in clinical studies. When compared with gem/nab-paclitaxel, it has a higher response rate which has corresponded to higher rates of R0 resection. This is why mFOLFIRINOX has been selected by US cooperative groups to be the backbone in future neo-adjuvant protocols in PDAC. Furthermore, the importance of response rates in the neo-adjuvant setting is of greater importance than in the metastatic setting as many of these patients are being treated with curative intent. Our institutional standard is to use this regimen in all patients who are candidates for neo-adjuvant chemotherapy. Some of the most recent data with Pembolizumab in GI malignancies has suggested that it can be administered safely and has clinical activity when combined with chemotherapy in metastatic gastric cancer Additionally, safety of Nivolumab has been well established in combination with FOLFOX or FOLFIRI in several clinical trials.

We hypothesize that preoperative treatment with chemotherapy and Nivolumab will deplete the stroma, increase intratumoral perfusion (and pathological response rate) without increased risk of post-operative complications in patients with borderline resectable PDAC. We will address this by (1a) investigating the feasibility and safety of preoperative treatment with the combination of Nivolumab and mFOLFIRINOX on the risk of post-operative clinically relevant pancreatic surgery toxicities ie fistula formation (expected to be <11%)(1b) evaluating pathologic complete response after neoadjuvant Nivolumab and FOLFIRINOX treatment. We will

	also investigate (3) the effects of this combination on CD8 infiltration and tumor microenvironment proliferation and apoptosis.
**STUDY DESIGN**	We will be conducting a Phase I/II study investigating Nivolumab in combination with mFOLFIRINOX in patients with borderline resectable PDAC. While identifying upfront resectable PDAC is fairly simple, there are multiple definitions of borderline resectable PDAC, including the MD Anderson definition and the criteria developed during the Consensus Conference sponsored by the American Hepato-Pancreato-Biliary Association, Society of Surgical Oncology, and Society for Surgery of the Alimentary Tract. Borderline resectable PDAC cases will be identified per the definition developed in the currently running inter-group pilot trial for borderline resectable pancreatic cancer (NCT01821612).<br><br>Eligibility will include patients with ECOG PS 0-1 who have normal hepatic and renal function and have resectable or borderline resectable PDAC. Patients with locally advanced PDAC or metastatic PDAC are excluded.<br><br>This trial will be conducted in two parts. In **Part I (6-12 patient safety lead in period)**, patients will be enrolled in cohorts of 4-6 patients to determine safety. Because Nivolumab has been dosed at its full dose with FOLFOX and FOLFIRI, we expect no additional safety signals will be revealed. But, additional monitoring for cytopenias and GI toxicities will occur in the first 6-12 patients. If there is an unacceptable level of GI toxicities or increased rates of expected Nivolumab toxicities, the study will be closed. Ongoing trials with several new agents in development have shown full doses of these agents do not increase toxicities of mFOLFIRINOX.<br><br>In **Part II**, an additional 21 patients will be enrolled, and will begin neoadjuvant therapy with Nivolumab and mFOLFIRINOX (mFFX). Reassessments will be as in part 1, but less frequent monitoring will be required once safety has been assured.<br><br>For the safety objectives in all patients, we will be monitoring the classical adverse events associated with all chemotherapy and Nivolumab. But because these patients will be treated in the neoadjuvant setting, additional importance will be on the rates of post-operative complications, with an emphasis on clinically relevant surgical complications per the standard clinical guidelines. The most common of which are the formation of pancreatic fistulas which often present with abdominal pain, leukocytosis and fever (temperature >100.4 degrees). Diagnostic work-up of pancreatic fistula will be with CT abdomen with contrast, which has a

	sensitivity of 63% and specificity of 83% for detecting pancreatic fistula. We will also track other relevant post-operative complications such as delayed wound healing, development of wound infections.<br><br>In all patients, pre-treatment EUS-guided core biopsies of the pancreatic tumor and CA 19-9 levels will be obtained. Subsequently, patients will be started on treatment with Nivolumab and mFOLFIRINOX given every 4 weeks for 12 weeks. Nivolumab will be given in the standard q4 week regimen 3 times in the first 12 weeks. To evaluate the disease response to treatment, CA 19-9 levels will be checked monthly and restaging CT chest, abdomen and pelvis will be obtained every 12 weeks. If the patients are found to have resectable disease, patients will be taken to an attempt at curative surgery. If at 12 weeks, the imaging shows stable disease but the patients are still not considered resectable by standard criteria, the chemotherapy will be continued for an additional 6 cycles (3 additional doses of Nivolumab) whereby restaging will be performed again. At the completion of 6 cycles of therapy (6 doses of Nivolumab), restaging CT scans will be obtained to determine resectability. If there is disease progression at any point in the study, patients will be taken off of study and alternative treatments will be offered. If the patients are able to have successful surgeries, tissue analyses will be performed on the resected pancreatic tumor. For those patients who are found to have unresectable disease in the operating room, an intraoperative core biopsy of the pancreatic tumor will be obtained for tissue analyses. No patients will receive more than 6 cycles (6 months) of neo-adjuvant therapy on this protocol. Postoperatively, patients will receive further (adjuvant) chemotherapy, as per investigator discretion typically consisting of gemcitabine +/- capecitabine or nab-paclitaxel, at the discretion of the treating provider.<br><br>The tissue analyses will include an assessment of the rates of pathological responses when compared to historical controls. Additionally, samples will be compared for the effects on T-cell infiltration in the tumors, PD1 expression levels and the effects on markers of the tumor microenvironment. If limited tissue sample is obtained via the core biopsies, the priority of tissue analysis will be as follows: (1) fixed in formalin for H&E and IHC (PDL1, CD8, PD1, PDL2); (2) fixed in OCT such that IHC with difficult antibodies can be done (to potentially obtain mRNA or DNA). The IHC studies will be done at the UCLA Jonsson Comprehensive Cancer Center Immunohistochemistry and Molecular Pathology Core, in collaboration with Dr. Cauis Radu in the tumor immunology group. The tissue analyses of the biopsy and surgical specimens will

be done by a GI pathologist, Dr. David Dawson, who is the co-director of surgical pathology and specializes in pancreatic pathology.

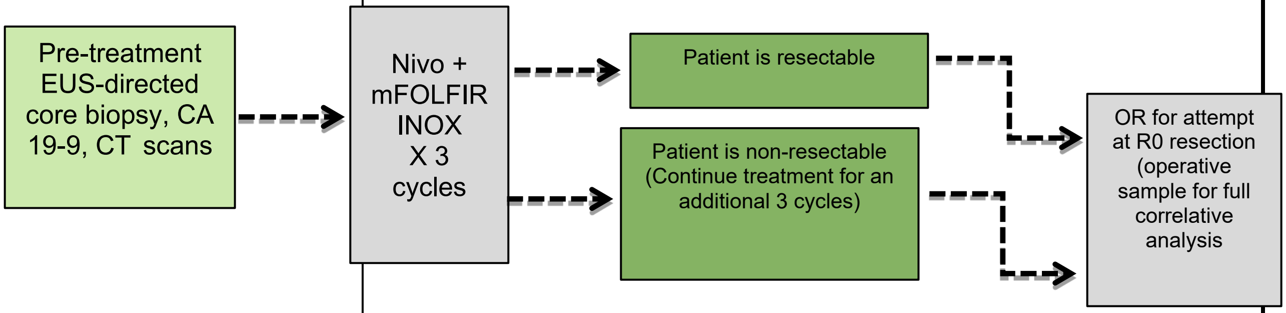

**PRIMARY OBJECTIVES**	To evaluate the safety, tolerability and pathologic complete response rate (pCR) in patients with borderline resectable pancreas adenocarcinoma treated with neoadjuvant mFOLFIRINOX and Nivolumab.
**SECONDARY OBJECTIVES**	To evaluate early efficacy measured by percent change of CA 19-9 response rate, R0 resection rate, overall response rate (ORR) and disease free survival (DFS).
**EXPLORATORY OBJECTIVES**	1) To evaluate the effects of Nivolumab on the effects of tumor-stromal cells comparing pre-treatment core biopsies with operative specimens and on the effects on T cell infiltration, PD1 expression and the tumor micro-environment<br><br>2) To establish PDX models from post treatment surgical specimens as a model for further study
**NUMBER OF SUBJECTS**	The required number of post-operative patients is 25 evaluable patients for the study. We expect to enroll 36 patients total for required sample size. We will enroll 6-12 patients in the first phase of the trial. 21 patients will be enrolled in the expansion cohort in the second phase.
**SUBJECT SELECTION CRITERIA**	**Inclusion Criteria**<br><br>- Greater than or equal to 18 years old.<br>- Histologically confirmed pancreatic adenocarcinoma<br>- Borderline resectable disease, as defined below<br>- Performance status of ECOG of 0-1<br>- Therapy naïve<br>- Adequate organ function including:

- Bone marrow: ANC ≥1500/mm3, platelets ≥100,000/mm3 and hemoglobin ≥ 9 g/dL
- Hepatic: Serum total bilirubin ≤1.5 x upper limit of normal (ULN), ALT (SGPT) and AST (SGOT) ≤ 2.5 x ULN, alkaline phosphatase ≤ 2.5X ULN.
- Renal: Serum creatinine (sCr) ≤ 1.5 x ULN, or creatinine clearance (Ccr) ≥ 40 mL/min as calculated by the Modified Cockcroft-Gault formula.

- Peripheral neuropathy < grade 2

Borderline Resectable is defined as: Presence of any one or more of the following on CT: (1) An interface between the primary tumor and the superior mesenteric vein or portal vein (SMV-PV) measuring ≥ 180° of the circumference of the vessel wall. (2) Short-segment occlusion of the SMV-PV with normal vein above and below the level of obstruction that is amenable to resection and venous reconstruction (3) Short segment interface (of any degree) between tumor and hepatic artery with normal artery proximal and distal to the interface that is amenable to resection and reconstruction. (4) An interface between the tumor and SMA measuring < 180º of the circumference of the vessel wall.

Exclusion Criteria:

- Age younger than 18 years old
- Locally advanced (clearly unresectable) or metastatic disease
- Known status of HIV which is not well-controlled at the time of study eligibility
- Untreated Hepatitis B infection
- Active infection or antibiotics within 48 hours prior to study
- Currently active second primary malignancy or history of malignancy less than 5 years prior to the time of study eligibility (Patients with history of skin cancers excluding melanoma will be eligible for participation).
- Serious medical comorbidities such as New York Heart Association Class III/IV cardiac disease, uncontrolled cardiac arrhythmias, myocardial infarction over the past 12 months.
- Known, existing uncontrolled coagulopathy. Patients who have had a venous thromboembolic event (e.g., pulmonary embolism or deep vein thrombosis) requiring anticoagulation are eligible IF: they are appropriately anticoagulated and have not had a Grade 2 or greater bleeding episode in the 3 weeks before Day 1.

-Any prisoners or people compulsory detained

- Known pregnancy, nursing women or positive pregnancy test.
- Any condition that would preclude informed consent, consistent follow-up and compliance for the study. participation.

**TEST PRODUCT, DOSE, AND ROUTE OF ADMINISTRATION**	FOLFIRINOX as is standard of care for the treatment of borderline resectable pancreatic cancer as per NCCN guidelines and at UCLA defined as:<br>1. 5-FU 2400 mg/m^2 over 48 hours<br>2. Oxaliplatin 85 mg/m^2 every 2 weeks<br>3. Irinotecan 150 mg/m^2 every 2 weeks<br>4. Prophylactic G-CSF (Peg-filgastrim)<br><br>This regimen has several differences from classical FOLFIRINOX which include: No bolus 5-FU, lower doses of irinotecan, and prophylactic G-CSF. Several studies have shown that this regimen can be given safely in the neo-adjuvant setting with an expected R0 resection in borderline pancreas cancer of 35%.<br><br>In combination with the chemotherapy, Nivolumab (480 mg) IV over 60 minutes will be given every 4 weeks to coincide with administration of chemotherapy so Nivolumab will be given every 28 days for a minimum of 3 and a maximum of 6 cycles.
**DURATION OF SUBJECT PARTICIPATION AND DURATION OF STUDY**	Subjects will be in the study for up to 6 months.<br>**Screening:** up to 21 days<br><br>**Treatment:** 4-6 months of chemotherapy followed by surgical resection if the patient's tumor is deemed resectable post-therapy.<br>-Treatment will continue until:<br>**a.** Disease progression<br>**b.** Unacceptable adverse event(s)<br>**c.** Treatment delay >2 weeks due to delayed recovery from treatment-related toxicity; or treatment delay of one agent for >4 weeks from time of last scheduled dose.<br>**d.** Patient decision to withdraw from the study.<br><br>**Follow-up:** Patients will be followed after completion of study until death, by either in-person evaluation or telephone contact by study personnel at 2-3 month intervals (or more frequently as indicated by their disease state).
**CONCOMITANT MEDICATIONS**	**Medications Allowed:**<br>-Medications used for supportive therapy such as anti-emetics, anti-diarrheals, analgesics, bisphosphonates and antibiotics are allowed as indicated.<br><br>**Medications Prohibited:**<br>-Patients may not be on any therapy for their pancreatic cancer other than the current study's treatment drugs.

**PRIMARY ENDPOINTS**	The incidence of pancreatic fistula within the 7-day post-operative period after neoadjuvant treatment with mFOLFIRINOX and nivolumab.<br><br>Rate of pathologic complete response after neoadjuvant treatment with mFOLFIRINOX and nivolumab.
**SECONDARY ENDPOINTS**	Percent change of CA 19-9 response rate<br>R0 resection rate<br>Overall response rate (ORR)<br>Disease-free survival (DFS).
**OTHER EVALUATIONS**	Tissue analyses pre-treatment and post-treatment with standard IHC staining: 1) To determine the changes in immune cell infiltrates and cancer cell IFNγ signaling in response to FOLFIRINOX and Nivolumab treatment.2) To map the signaling and metabolomic changes in PDAC cancer cells that respond to PD1 blockade. If limited tissue sample is obtained via the core biopsies, the priority of tissue analysis will be as follows: (1) fixed in formalin for H&E and IHC (CD8, CD3); (2) fresh tissue samples for transcriptomic, proteomic, phosphoproteomic, and metabolomic studies, generated from fresh tumors of "Responder" patients to generate a multi-dimensional integrated map of the key changes stimulated by nivolumab in tumor cells
**SAFETY EVALUATIONS**	Post-operative complication of pancreatic fistula will be monitored per the standard clinical guidelines. Common clinical presentations of clinically relevant pancreatic fistula include abdominal pain, leukocytosis and fever (temperature >100.4 degrees). Diagnostic work-up of pancreatic fistula will be with CT abdomen with contrast, which has a sensitivity of 63% and specificity of 83% for detecting pancreatic fistula [33]. The pancreatic fistulas will be categorized into grades A, B or C as previously reported [34]. Clinically relevant pancreatic fistulas are categorized as grade B or C. Depending on the grade of pancreatic fistula, patients will be treated as indicated with conservative treatment options including bowel rest, medications and sometimes with interventional radiology assistance [34]. We will also track other relevant post-operative complications such as delayed wound healing, development of wound dehiscence or wound infection.
**STATISTICS**<br><br>**Primary Analysis Plan**	One of the primary endpoints of the study is to evaluate development of clinically relevant pancreatic fistula within the 7-day post-operative period after neoadjuvant treatment with mFFX and nivolumab. The co-primary endpoint will evaluate the pathologic complete response after neoadjuvant treatment with mFFX and nivolumab. Descriptive statistics with frequency and proportion will be used to evaluate the primary endpoint. We assume that the proportion of patients who may experience post-operative

<table>
<tr><td></td><td>complications is less than 11%. With a sample size of 25 evaluable patients, we will be able to estimate the 90% CI to be [4.5%-19.89%] or 95% CI to be [3.36%-23.07%]. We assume that about 30-35% of patients will not be eligible for post-surgery evaluation, we will enroll 36 patients in the study in order to achieve the targeted sample size of 25 evaluable patients.

In order to ensure patient safety, this trial includes early stopping rules in the event of unexpected postoperative complications such as development of pancreatic fistula. Two interim safety assessments will be performed when first 7 and 15 evaluable patients are enrolled in the study. If three out of first 7 patients or four out of first 15 patients develop pancreatic fistula, this trial will be stopped. The early stopping rules of declaring the trial unsafe based on assumed postoperative complications of < 11% and type I alpha of 10% are described in Table 1.

Early Stopping Criteria (assuming %complications < 11%)

<table>
<tr><td># of evaluable patients enrolled</td><td>7</td><td colspan="2">15</td></tr>
<tr><td>Alpha (Type I error rate)</td><td>10%</td><td>10%</td><td>5%</td></tr>
<tr><td># of post-op complications</td><td>3</td><td>4</td><td>5</td></tr>
</table>
</td></tr>
<tr><td>Secondary Analysis Plan</td><td>Secondary endpoints include early efficacy as measured by percent change of CA 19-9 response rate, R0 resection rate, overall response rate (ORR) and disease free survival (DFS). Descriptive statistics with frequency and proportion will be used to analyze the CA19-9 response rate and ORR. Kaplan-Meier methods will be used to analyze DFS with median and 95% CI.</td></tr>
<tr><td>Correlative Studies Analyses</td><td><u>Tissue Analyses:</u>
We will examine the tissue from baseline to post-therapy at the time of surgery or at the time of progression. We will use descriptive statistics and graphical displays to compare the percent change in stromal depletion overall and to describe the association with cell proliferation and death. In addition, we will graphically explore the percent change in stromal depletion for patients who undergo surgery compared to those who have disease progression.</td></tr>
<tr><td>Rationale for Number of Subjects</td><td>With a sample size of 25 evaluable patients, we will be able to estimate the 90% CI to be [4.5%-19.89%] or 95% CI to be [3.36%-23.07%].</td></tr>
</table>

Table of Contents

Protocol Signature Page **3**
Table of Contents **13**
1 BACKGROUND **17**
1.1 Pancreatic Cancer: Overview of Resectable and Borderline Resectable Disease 17
1.2 Targeting the Tumor Microenvironment: Nivolumab 17
1.3 Pancreatic Fistula Definition 18
1.4 Background on the Compounds 18
1.4.1 FOLFIRINOX for Pancreatic Cancer 18
2 STUDY RATIONALE **20**
3 STUDY OBJECTIVES **20**
3.1 Primary Objectives 20
3.2 Secondary 20
3.3 Exploratory Objectives, Other Assessments 20
4 STUDY DESIGN **20**
4.1 Study Overview 20
5 CRITERIA FOR EVALUATION **22**
5.1 Primary Endpoints 22
5.2 Secondary Endpoints 22
5.3 Safety Evaluations 22
5.4 Correlative Endpoints 22
6 SUBJECT SELECTION **22**
..... **22**
6.1 Inclusion Criteria 22
6.2 Exclusion Criteria 23
7 CONCURRENT MEDICATIONS **24**
7.1 Allowed Medications and Treatments 24
8 STUDY TREATMENTS **24**
8.1 Study Agent Administration 24
8.2 Formulation of Study Agents 24
8.2.1 Oxaliplatin ..... **Error! Bookmark not defined.**
8.2.2 Irinoteca ..... **Error! Bookmark not defined.**
8.2.3 5-FU ..... **Error! Bookmark not defined.**
8.2.4 Rules for Dose Omissions and Modified Schedules 30
8.3 Study Drug Accountability 42
9 STUDY PROCEDURES AND GUIDELINES **42**
9.1 Clinical Assessments 43
9.1.1 Concomitant Medications 43
9.1.2 Demographics 43
9.1.3 Medical History 43
9.1.4 Physical Examination 43
9.1.5 Vital Signs 43

9.1.6 Adverse Events ... 43
9.2 Clinical Laboratory Measurements ... 43
9.2.1 Hematology ... 43
9.2.2 Blood Chemistry Profile ... 43
9.2.3 Pregnancies ... 43
9.2.4 Pregnancy Test ... 44
9.3 Tumor marker ... 44
9.4 Research Tissue Analyses ... 44
9.5
10 EVALUATIONS BY VISIT ... 44
11 ADVERSE EXPERIENCE REPORTING AND DOCUMENTATION ... 46
11.1 Serious Adverse Experiences (SAE) ... 47
11.1.1 Serious Adverse Experience Reporting ... 48
11.1.2 Reporting of Serious Adverse Events to BMS ... 50
11.2 Data and Safety Monitoring Committee (DSMC) Contacts ... 54
12 DISCONTINUATION AND REPLACEMENT OF SUBJECTS ... 54
12.1 Early Discontinuation of Study Drug ... 54
12.2 Withdrawal of Subjects from the Study ... 55
12.3 Replacement of Subjects ... 55
13 PROTOCOL VIOLATIONS ... 55
14 STATISTICAL METHODS AND CONSIDERATIONS ... 55
14.1 Data Sets Analyzed ... 55
14.2 Demographic and Baseline Characteristics ... 56
14.3 Analysis of Primary Endpoint ... 56
14.4 Analysis of Secondary Endpoint ... 56
14.5 Analyses of the Correlative Studies ... 56
15 DATA COLLECTION, RETENTION AND MONITORING ... 57
15.1 Data Collection Instruments ... 57
15.2 Data Management Procedures ... 57
15.3 Data Quality Control and Reporting ... 57
15.4 Archival of Data ... 58
15.4.1 Availability and Retention of Investigational Records ... 58
15.4.2 Monitoring ... 58
16 ADMINISTRATIVE, ETHICAL, REGULATORY CONSIDERATIONS ... 59
16.1 Protocol Amendments ... 59
16.2 Institutional Review Boards and Independent Ethics Committees ... 60
16.3 Informed Consent Form ... 60
16.4 Publications ... 62
16.5 Investigator Responsibilities ... 62
18 References ... 63

List of Tables

Table 1. Dose Reductions for FOLFIRINOX 30

Table 2. Dose Reductions for Nivolumab .... **Error! Bookmark not defined.**

Table 3. Dose Modifications for Hematologic Toxicities at the Start of Each Cycle or Within a Cycle 30

Table 4. Dose Modifications for Non-Hematologic Toxicity 31

Table 5. Timeline of Clinical Assessment, Studies and Treatment 45

Table 6. AE Severity Grading 47

Table 7. AE Relationship to Study Drug 47

List of Abbreviations

**AE**	adverse event
**ALT**	alanine aminotransferase
**AST**	aspartate aminotransferase
**BUN**	blood urea nitrogen
**CFR**	Code of Federal Regulations
**CRF**	case report form
**DMC**	Data Monitoring Committee
**DSMB**	Data Safety Monitoring Board
**FDA**	Food and Drug Administration
**GCP**	Good Clinical Practice
**HIPAA**	Health Insurance Portability and Accountability Act of 1996
**ICF**	informed consent form
**ICH**	International Conference on Harmonisation
**IEC**	Independent Ethics Committee
**IRB**	Institutional Review Board
**IV**	intravenous
**mEq**	milliequivalent
**PI**	Principal Investigator
**PK**	pharmacokinetic
**SAE**	serious adverse experience

1 BACKGROUND

1.1 Pancreatic Cancer: Overview of Resectable and Borderline Resectable Disease

Pancreatic ductal adenocarcinoma (PDAC) continues to be a challenging cancer to treat secondary to its advanced stage at the time of diagnosis, its aggressive behavior and its resistance to treatment[1]. Only a minority of patients present with localized, non-metastatic disease and may potentially represent surgical candidates. Such patients may be divided into two categories: those who have clearly resectable disease at initial diagnosis (herein referred to as upfront resectable), and those with borderline resectable pancreatic cancer (BRPC). BRPC represents an increasingly recognized, distinct clinical entity characterized by primary tumor involving surrounding vasculature with a high risk for margin-positive resection if resected de novo [2].

Neoadjuvant therapy is often considered for patients with both resectable and borderline resectable disease to eradicate any micrometastatic disease, to identify suitable candidates most likely to benefit from surgery, and to improve the likelihood of margin-negative (R0) resection. However, consensus regarding the optimal neoadjuvant approach, including the respective roles, necessity, sequencing, and duration of neoadjuvant chemotherapy and chemoradiation (chemoRT), is lacking (reviewed in [3]). The neoadjuvant setting also represents an ideal setting to evaluate novel therapies to assess their on-target/pharmacodynamic effects, given that the majority of patients will ultimately be taken to surgery and be able to provide abundant tumor material for analyses.

1.2 Targeting the Tumor Microenvironment: Nivolumab

The PDAC tumor microenvironment is complex and consists of multiple components including pancreatic stellate cells (PSC), endothelial cells, immune cells and fibroblasts with an extensive paracrine and autocrine network residing in a stiff extracellular matrix [4-6]. The desmosplastic reaction that is characteristic of the PDAC stroma [6, 7] has been associated with increased cell proliferation and migration leading to metastasis in PDAC [8-11], while it disturbs the pancreatic tissue architecture by changing the layout of blood and lymphatic vessels [7, 12].

Immunotherapy has emerged as a therapeutic approach that offers effective and durable treatment options for subsets of patients with various types of cancer. However, these successes have not manifested similar benefits for PDAC patients mostly due to a lack of pre-existingT-cell immunity and/or a highly immunosuppressive tumor microenvironment (TME).Considering the emerging role of the TME, the combination of checkpoint blocking antibodies with agents that target the inhibitory effects of the TME could lead to better responses in tumors historically resistant to checkpoint blocking antibody approaches. The addition of chemotherapy could further potentiate the anti-tumor effects of immunotherapy approaches by reducing the tumor burden, exposing antigens and directly affecting the immunosuppressive TME compartment.

Until recently, chemotherapy was thought to have negative effects on the immune system by reducing the lymphocyte numbers and inhibiting the expansion of activated lymphocytes. In reality, chemotherapy can promote the anti-tumor effects of the immune system by decreasing tumor burden, promoting immunogenic cell death, and interfering with the immunosuppressive cancer cells.

Based on the different MOA of nivolumab and FOLFIRINOX (FFX), and data from the combination of nivolumab and other FOLFOX and FOLFIRI chemotherapy regimens, the risk for overlapping toxicity is expected to be low.

We selected mFFX as the chemotherapy backbone due to the safety and efficacy profile reported in clinical studies. Several trials, have recently shown that m FFX has the highest efficacy reported in PDAC and can be given safely before surgery [13]. This study in a similar group of patients demonstrated a median overall survival of 37.7 months and 2-year survival of 56%. In addition, R0 resection was seen in 65% of patients. The role of the PDAC tumor microenvironment in formation of a tumor suppressive environment and increasing metastatic potential makes it an important target for therapeutic intervention. We hypothesize that preoperative treatment with nivolumab will open the TME to the effects of more cytotoxicity allowing for increased intratumoral perfusion (and hopefully drug delivery) without increased risk of post-operative complication of pancreatic fistula formation in patients with borderline resectable PDAC. We will address this by investigating the effect of preoperative treatment with the combination of nivolumab and mFFX on the risk of post-operative clinically relevant pancreatic fistula formation evaluating pathologic complete response after neoadjuvant nivolumab and mFFX treatment. In addition, we will investigate the degree of the TME and the effects of nivolumab on the TME.

1.3 Pancreatic Fistula Definition

Pancreatic fistula is the "failure of healing/sealing of pancreatic-enteric anastomosis or it may represent a parenchymal leak not directly related to an anastomosis". It is considered to be a common post-operative complication following pancreatic surgery with a frequency ranging from 5-30% [14-21]. Development of pancreatic fistula may affect timely initiation of adjuvant therapy following surgery due to possible longer hospital stays. A universally accepted Definition of pancreatic fistula was developed by the International Study Group on Pancreatic Fistula (ISGPF) in 2005 and classified pancreatic fistulas to 3 grading systems; A, B and C[22]. According to this definition, pancreatic fistula is defined as "any appreciable drainage from an operatively placed drain with an amylase content greater than 3 times the upper limit of normal serum amylase level measure on, or after, postoperative day 3"[23]. Grade A fistulas are diagnosed biochemically with elevated amylase but do not have any associated symptoms and therefore are not clinically relevant. Grade B and C fistulas on the other hand are symptomatic fistulas, which require diagnostic evaluation and therapeutic interventions. Patients often present with "abdominal pain, fever, nausea and intolerance to oral intake" and diagnostic imaging studies may show peripancreatic fluid collection[24]. The degree of intervention is more intense with grade C fistulas as these patients are often in critical condition with possible sepsis and organ dysfunction. While patients with grade B pancreatic fistulas may be treated with antibiotics and percutaneous drainage, patients with grade C pancreatic fistulas may require ICU admission, surgical re-exploration, antibiotics and somatostatin analogues. In the current study, we will be evaluating clinically relevant pancreatic fistulas, i.e. pancreatic fistulas graded as B or C, within in the first week following surgery.

1.4 Background on the Compounds

1.4.1 FFX for Pancreatic Cancer

The recent randomized controlled trial with FOLFIRINOX revealed improved response in overall and progression free survival over single-agent gemcitabine in metastatic pancreatic cancer and established this regimen as one of the standard options for patients with metastaic disease [25].More recently this regimen has been investigated in the neoadjuvant, locally advanced and adjuvant settings [26]. In all of

these settings, this regimen was shown to be active, tolerable and able to produce efficacy outcomes not seen with other chemotherapy combinations.

1.4.2 Nivolumab

Cancer immunotherapy rests on the premise that tumors can be recognized as foreign rather than as self and can be effectively attacked by an activated immune system. An effective immune response in this setting is thought to rely on immune surveillance of tumor antigens expressed on cancer cells that ultimately results in an adaptive immune response and cancer cell death.

Meanwhile, tumor progression may depend upon acquisition of traits that allow cancer cells to evade immuno-surveillance and escape effective innate and adaptive immune responses. Current immunotherapy efforts attempt to break the apparent tolerance of the immune system to tumor cells and antigens by either introducing cancer antigens by therapeutic vaccination or by modulating regulatory checkpoints of the immune system. T-cell stimulation is a complex process involving the integration of numerous positive as well as negative co-stimulatory signals in addition to antigen recognition by the T-cell receptor (TCR). Collectively, these signals govern the balance between T-cell activation and tolerance.

PD-1 is a member of the CD28 family of T-cell co-stimulatory receptors that also includes CD28, CTLA 4, ICOS, and BTLA. PD-1 signaling has been shown to inhibit CD28-mediated upregulation of IL-2, IL-10, IL-13, interferon-γ (IFN-γ) and Bcl-xL. PD-1 expression has also been noted to inhibit T-cell activation and expansion of previously activated cells. Evidence for a negative regulatory role of PD-1 comes from studies of PD-1 deficient mice, which develop a variety of autoimmune phenotypes [27]. PD1/PD-L1 interactions may also indirectly modulate the response to tumor antigens through T-cell/APC interactions. Therefore, PD-1 engagement may represent one means by which tumors evade immuno-surveillance and clearance [28]. Blockade of the PD-1 pathway by nivolumab has been studied in a variety of preclinical in vitro assays, and anti-tumor activity using a murine analog of nivolumab has been shown in a number of immunocompetent mousecancer models.

In the US, nivolumab has been approved for the treatment of melanoma, NSCLC, RCC, MSI-H cancers, metastatic gastric cancer and classical Hodgkin lymphoma. Nivolumab is currently being evaluated extensively across a wide range of solid tumors and hematological malignancies. These findings provided the rationale for expanding the evaluation PD-1 pathway blockade in combination with novel immunotherapy agents in clinical studies.

Nivolumab is a fully human, immunoglobulin G4 (IgG4) [kappa] isotype monoclonal antibody that binds to PD-1 with nanomolar affinity (dissociation constant [Kd], 3.06 nM) with a high degree of specificity. In vitro, nivolumab (BMS-936558) binds to PD-1 with high affinity (EC50 0.39-2.62 nM), and inhibits the binding of PD-1 to its ligands PD-L1 and PD-L2 (IC50 □ 1 nM). Nivolumab binds specifically to PD-1 and not to related members of the CD28 family such as CD28, ICOS, CTLA-4, and BTLA. Blockade of the PD-1 pathway by nivolumab results in a reproducible enhancement of both proliferation and IFN-γ release in the mixed lymphocyte reaction (MLR). Using a cytomegalovirus (CMV) re-stimulation assay with human peripheral blood mononuclear cells (PBMC), the effect of nivolumab on antigen specific recall response indicates that nivolumab augmented IFN-γ secretion from CMV specific memory T cells in a dose-dependent manner versus isotype-matched control. In vivo blockade of PD-1 by a murine analog

of nivolumab enhances the anti-tumor immune response and result in tumor rejection in several immunocompetent mouse tumor models (MC38, SA1/N, and PAN02).

Details of the in vitro and in vivo nonclinical pharmacology studies conducted to support the development of nivolumab can be found in the Nivolumab IB. The safety of the combination will be carefully monitored in the planned clinical trial. The overall safety experience with nivolumab, as either monotherapy or in combination with other therapeutics, is based on experience in approximately 12,300 participants.

Nivolumab, alone or in combination with chemotherapy, is currently being tested for the treatment of advanced/metastatic PDAC. Preliminary results from Study CA209032 show that nivolumab, administered as monotherapy, was well tolerated in participants with advanced/metastatic PDAC. In addition, the combination of nivolumab and gemcitabine/abraxane is currently being tested as first line treatment for participants with advanced/metastatic PDAC (NCT02309177). The combination appears to be well tolerated with a safety profile similar to the individual components. As of February 2017, 20 participants are evaluable for efficacy. The preliminary ORR is 25% (5 out of 20 participants experience PR). In addition, 12 participants experienced prolonged, stable disease. Taken together, these data suggest that while nivolumab in monotherapy may not be sufficient to improve the anti-tumor activity in advanced pancreatic cancer, the combination of nivolumab with the appropriate chemotherapy may indeed improve anti-tumor activity in this setting.

1.4.3 STUDY RATIONALE

Because of its mechanism of action as mentioned above, we believe that nivolumab could have a role in the neoadjuvant setting for patients with borderline resectable pancreatic ductal adenocarcinoma. While this agent already has an established safety profile in combination with FFX, we wish to assess both its safety and efficacy in this preoperative context.

2 STUDY OBJECTIVES

2.1 Primary Objectives

- To evaluate development of clinically relevant pancreatic fistula in the post-operative period after neoadjuvant treatment with nivolumab and FOLFIRINOX (FFX).
- To evaluate pathologic complete response after neoadjuvant nivolumab and FOLFIRINOX (FFX).

2.2 Secondary

To evaluate early efficacy measured by percent change of CA 19-9 response rate, R0 resection rate, overall response rate (ORR) and disease free survival (DFS).

2.3 Exploratory Objectives, Other Assessments

- To determine degree of changes in the TME of nivolumab and m FFX on cell proliferation and apoptosis.

3 STUDY DESIGN

3.1 Study Overview

We will be conducting a Phase II study investigating nivolumab and FOLFIRINOX (FFX) in patients with borderline resectable PDAC at the Jonsson Comprehensive Cancer Center at UCLA.

This trial will be conducted in two parts. In Part I, pre-treatment EUS-guided core biopsies of the pancreatic tumor, CA 19-9 levels will be obtained for the first six-twelve patients enrolled. After enrollment, patients will be started on treatment with nivolumab and FOLFIRINOX (FFX) given bi-weekly. To evaluate the disease response to treatment, CA 19-9 levels will be checked monthly and restaging CT chest, abdomen and pelvis will be obtained every 12 weeks. If there is disease progression at any point in the study, patients will be taken off of study and alternative treatments will be offered. At the completion of 6 cycles of therapy, restaging CT scans will be obtained to determine resectability. If the patients are able to have successful surgeries, tissue analyses will be performed on the resected pancreatic tumor. If the patients are deemed to be surgical candidates but are found to have unresectable disease in the operating room, an intraoperative core biopsy of the pancreatic tumor will be obtained for tissue analyses.

In Part II, an additional 21 patients will be enrolled, and will begin neoadjuvant therapy with nivolumab and FOLFIRINOX (FFX).

For Specific Aim 1, we will be monitoring the post-operative complication of clinically relevant pancreatic fistula within one week of surgery per the standard clinical guidelines as noted above. Common clinical presentations of pancreatic fistula include abdominal pain, leukocytosis and fever (temperature >100.4 degrees). Diagnostic work-up of pancreatic fistula will be with CT abdomen with contrast, which has a sensitivity of 63% and specificity of 83% for detecting pancreatic fistula [33]. The pancreatic fistulas will be categorized into grades A, B or C as previously reported [34]. Our study will be investigating only clinically relevant pancreatic fistulas, i.e. grades B or C. Depending on the grade of pancreatic fistula, patients will be treated as indicated with conservative treatment options including bowel rest, antibiotics, somatostatin analogues and percutaneous drainage or surgical re-exploration[34]. We will also track other relevant post-operative complications such as delayed wound healing, development of wound dehiscence or wound infection.

The tissue analyses will include review of the immunohistochemical (IHC) stains for PDL1, PD1, CD8, CD3, CD6 and a comprehensive human pan-immune cell CyTOF panel (32 antibodies for characterization of B lymphocytes, T lymphocytes, NK cells, MDSCs, iNKT, and gamma/delta T cells) will be studied. If limited tissue sample is obtained via the core biopsies, the priority of tissue analysis will be as follows: (1) fixed in formalin for H&E and IHC (Ph-H3; CC3/Tunel; HA binding); (2) fixed in OCT such that IHC with difficult antibodies can be done (to potentially obtain mRNA or DNA). The IHC studies will be done at the UCLA Pathology Department and the tissue analyses of the biopsy and surgical specimens will be done by study pathologist, Dr. David Dawson, who is the co-director of GI pathology and specializes in pancreatic and liver pathology.

Cycle 1 and onward

Days 1, 15 out of a 28-day cycle	Nivolumab 480 mg IV q 4 weeks<br>FOLFIRINOX (q 2 weeks) as is standard of care for the treatment of borderline resectable pancreatic cancer as per NCCN guidelines and at UCLA defined as:<br>1. 5-FU 2400 mg/m^2 over 48 hours<br>2. Oxaliplatin 85 mg/m^2 every 2 weeks<br>3. Irinotecan 150 mg/m^2 every 2 weeks

	4. Prophylactic G-CSF (Peg-filgastrim)<br><br>Dexamethasone 10 mg PO 1-2 hours pre-chemo

- mFFX will be administered 1 hour post-nivolumab
- Neoadjuvant therapy will be three-six 28-day cycles with CT chest, abdomen and pelvis scans and CA 19-9 measurements every 8 weeks. Patients will be taken off of the study if there is any evidence of disease progression.
- After a maximum of six cycles of neoadjuvant therapy, patients will be evaluated for surgical resection depending on the CT scan findings obtained
    - If they have resectable diseasethey will have an attempt at surgery. If patients have successful resection, they will receive either three or 0 additional cycles of adjuvant mFFX. If they are found to have unresectable disease in the operating room, an intra-operative core biopsy will be obtained.
    - If they have unresectable disease on the restaging scans following six cycles of therapy, they will be taken off of the study.

4 CRITERIA FOR EVALUATION

4.1 Primary Endpoints

- Clinically relevant pancreatic fistula in the post-operative period after neoadjuvant treatment with nivolumab and FOLFIRINOX (mFFX) chemotherapy.
- Pathologic complete response after neoadjuvant FOLFIRINOX (mFFX) chemotherapy

4.2 Secondary Endpoints

- Percent change of CA 19-9 response rate,
- R0 resection rate
- Overall response rate (ORR)
- Disease free survival (DFS)

4.3 Safety Evaluations

- Safety and tolerability (adverse events, categorized and graded according to NCI CTC v. 4.0)
- Other relevant post-operative complications, including:
    - delayed wound healing
    - wound dehiscence
    - wound infection

4.4 Correlative Endpoints

1. To determine the changes in immune cell infiltrates and cancer cell IFNγ signaling in response to FOLFIRINOX and Nivolumab treatment.

2. To map the signaling and metabolomic changes in PDAC cancer cells that respond to IFNγ.

5 SUBJECT SELECTION

5.1 Inclusion Criteria

1. Greater than or equal to 18 years old.
2. Histologically confirmed pancreatic adenocarcinoma
3. One of the following:

a. **Borderline resectable disease**. There are multiple definitions of borderline resectable PDAC including the MD Anderson definition [32] and the criteria developed during the Consensus Conference sponsored by the American Hepato-Pancreato-Biliary Association, Society of Surgical Oncology, and Society for Surgery of the Alimentary Tract [33]. Borderline resectable PDAC cases will be identified per the definition developed in the currently running inter-group pilot trial for borderline resectable pancreatic cancer (NCT01821612). Per this trial, borderline resectable PDAC is defined as the presence of any one or more of the following on CT:
    i. An interface between the primary tumor and the superior mesenteric vein or portal vein (SMV-PV) measuring ≥ 180° of the circumference of the vessel wall
    ii. Short-segment occlusion of the SMV-PV with normal vein above and below the level of obstruction that is amenable to resection and venous reconstruction
    iii. Short segment interface (of any degree) between tumor and hepatic artery with normal artery proximal and distal to the interface that is amenable to resection and reconstruction.
    iv. An interface between the tumor and SMA measuring < 180º of the circumference of the vessel wall.

4. Performance status of ECOG of 0-1
5. Therapy naïve
6. Adequate organ function including:
7. Bone marrow: ANC ≥1500/mm^3, platelets ≥100,000/mm^3 and hemoglobin ≥ 9 g/dL
8. Hepatic: Serum total bilirubin ≤1.5 x upper limit of normal (ULN), ALT
9. (SGPT) and AST (SGOT) ≤ 2.5 x ULN, Alkaline phosphatase ≤ 2.5X ULN
10. Renal: Serum creatinine (sCr) ≤ 1.5 x ULN, or creatinine clearance (Ccr) ≥ 40 mL/min as calculated by the Modified Cockcroft-Gault formula.
11. Peripheral neuropathy < grade 2

5.2 Exclusion Criteria

1. Age younger than 18 years old
2. Locally advanced (clearly unresectable) or metastatic disease
3. Known status of HIV which is not well-controlled at the time of study eligibility
4. Untreated Hepatitis B infection
5. Active infection or antibiotics within 48 hours prior to study
6. Currently active second primary malignancy or history of malignancy less than 5 years prior to the time of study eligibility (Patients with history of skin cancers excluding melanoma will be eligible for participation).
7. Serious medical comorbidities such as New York Heart Association Class III/IV cardiac disease, uncontrolled cardiac arrhythmias, myocardial infarction over the past 12 months.
8. Known, existing uncontrolled coagulopathy. Patients who have had a venous thromboembolic event (e.g., pulmonary embolism or deep vein thrombosis) requiring anticoagulation are eligible IF: they are appropriately anticoagulated and have not had a Grade 2 or greater bleeding episode in the 3 weeks before Day 1.
9. Prior history of cerebrovascular accident or transient ischemic attack, or pre-existing carotid artery disease.
10. Known pregnancy, nursing women or positive pregnancy test. Requirement for WOCBP must have a pregnancy test every 4 weeks and WOCBP must have a negative serum or urine pregnancy

test (minimum sensitivity 25 IU/L or equivalent units of HCG) within 24 hours prior to the start of nivolumab
11. Any prisoners, or subjects who are compulsory detained are excluded
12. Any condition that would preclude informed consent, consistent follow-up and compliance for the study participation.

6 CONCURRENT MEDICATIONS

All subjects should be maintained on the same medications throughout the entire study period, as medically feasible, with no introduction of new chronic therapies.

6.1 Allowed Medications and Treatments

Medications Allowed:

- Medications used for supportive therapy such as anti-emetics, anti-diarrheals, analgesics, bisphosphonates and antibiotics are allowed as indicated.
- Thromboembolic events will be managed with anti-coagulation
- At the first occurrence of fever > 38.5C (regardless of neutrophil count), either oral levofloxacin (500 mg daily) or amoxicillin/clavulanate (500 mg tid) will be initiated.
- For febrile neutropenia, interrupt chemotherapy until fever resolves and ANC ≥ 1500 then resume treatment at a reduced dose.

Medications Prohibited:

- Patients may not be on any therapy for their pancreatic cancer other than the current study's treatment drugs.

7 STUDY TREATMENTS

7.1 Study Agent Administration

Each treatment cycle consists of 28 days as mentioned in Section 4.1.

Nivolumab 480 mg will be administered intravenously over a 60 minute period (+/- 20 minute window) on days 1 of every 28 day cycle.

5-FU 2400 mg/m2 IV The bolus will be administered over 10 minutes and the infucion will be administered in a pre-programmed pump over 46 hours.

Irinotecan 150 mg/m2 IV will be administered intravenously on days 1, 15 of each cycle over 90-120 minutes

Leucovorin 400 mg/m2 will be administered on days 1, 15 of each cycle over 120 minutes (concurrent with oxaliplatin)

Oxaliplatin 85 mg/m2 IV will be administered intravenously on days 1, 15 of each cycle over 120 minutes (concurrent with leucovorin)

7.2 Formulation of Study Agents

7.2.1 Nivolumab

7.2.1.1 Description

Nivolumab is a fully human immunoglobulin G4 (IgG4) monoclonal antibody that selectively inhibits programmed cell death-1 (PD-1) activity by binding to the PD-1 receptor to block the ligands PD-L1 and PD-L2 from binding.

7.2.1.2 Main Side effects (>10%):

- Cardiovascular: Edema (≤13%), peripheral edema (≤13%)
- Central nervous system: Fatigue (≤57%), malaise (≤46%), headache (17% to 23%), peripheral neuropathy (new onset and exacerbations: ≤14%; grade 3: <1%), dizziness (≤11%)
- Dermatologic: Skin rash (1% to 40%; immune-mediated: 9% to 16%), pruritus (10% to 28%), vitiligo (≤11%)
- Endocrine & metabolic: Hyperglycemia (42%), hyponatremia (16% to 41%), increased serum triglycerides (32%), hyperkalemia (≥10% to 30%), increased thyroid stimulating hormone level (≥10% to 26%), hypocalcemia (10% to 26%), increased serum cholesterol (21%), hypercalcemia (2% to 19%), thyroiditis (≤12% to 18%; including immune-mediated events), hypokalemia (16%), hypomagnesemia (14% to 16%), thyroid dysfunction (15%), hypothyroidism (≤12%; including immune-mediated events)
- Gastrointestinal: Diarrhea (1% to 37%), increased serum lipase (20% to 29%), decreased appetite (22% to 28%), nausea (20% to 28%), constipation (10% to 23%), abdominal pain (13% to 21%), vomiting (12% to 19%), increased serum amylase (>10% to 18%)
- Genitourinary: Urinary tract infection (2% to 17%)
- Hematologic & oncologic: Lymphocytopenia (27% to 42%; grade 3/4: ≤11%), anemia (26% to 40%; grade 3/4: 3% to 8%), leukopenia (11% to 38%; grades 3/4: ≤5%), neutropenia (13% to 37%; grade 3/4: 5%), thrombocytopenia (15% to 37%; grade 3/4: 2% to 3%)
- Hepatic: Increased serum aspartate aminotransferase (22% to 33%), increased serum alkaline phosphatase (10% to 33%), increased serum alanine aminotransferase (23% to 31%), increased serum bilirubin (11% to 13%)
- Immunologic: Graft versus host disease (20%; within 14 days of stem cell infusion), antibody development (11%; neutralizing: <1%)
- Neuromuscular & skeletal: Asthenia (≤57%), musculoskeletal pain (20% to 33%), back pain (21%), arthralgia (10% to 20%)
- Renal: Increased serum creatinine (12% to 42%)
- Respiratory: Upper respiratory tract infection (≥2% to 44%), cough (18% to 36%; includes productive cough), dyspnea (2% to 27%), bronchopneumonia (≤13%), pneumonia (≤13%), nasal congestion (11%)
- Miscellaneous: Febrile reaction (35%; events without an infectious cause that required steroids), fever (≤29%; may include tumor-associated fever), infusion-related reaction (≤14%)

1% to 10%:

- Cardiovascular: Pulmonary embolism (2% to 3%)
- Central nervous system: Neuritis (<10%), peripheral nerve palsy (peroneal: <10%)
- Dermatologic: Erythema (10%)

Endocrine & metabolic: Hyperthyroidism (3%; including immune-mediated events), adrenocortical insufficiency (1%; including immune-mediated events), increased gamma-glutamyl transferase

Gastrointestinal: Intestinal perforation (<10%), stomatitis (<10%), colitis (including immune-mediated events: ≤6%)

Hepatic: Hepatitis (immune-mediated: 2% to 3%)

Immunologic: Sjogren syndrome (<10%)

Infection: Sepsis (≥2%, systemic inflammatory response)

Neuromuscular & skeletal: Myopathy (<10%), rheumatism (spondyloarthropathy: <10%)

Renal: Acute renal failure (≥2%), nephritis (≤1%; immune-mediated), renal insufficiency (≤1%; immune-mediated)

Respiratory: Interstitial pulmonary disease (6%), pneumonitis (≤6%; including immune-mediated events), pleural effusion (1% to 5%), respiratory failure (≥2%)

7.2.1.3 How Supplied

Nivolumab (Opdivo) is supplied as a solution, Intravenous [preservative free]: 100 mg/10 mL (10 mL); [contains polysorbate 80]. Nivolumab injection is to be administered as an IV infusion through a 0.2-micron to 1.2-micron pore size, low-protein binding (polyethersulfone membrane) in-line filter at the protocol-specified doses and infusion times. It is not to be administered as an IV push or bolus injection. When the dose is based on patient weight (ie, mg/kg), nivolumab injection can be infused undiluted (10 mg/mL) or diluted with 0.9% Sodium Chloride Injection, USP or 5% Dextrose Injection, USP to protein concentrations as low as 0.35 mg/mL. When the dose is fixed (eg, 240 mg, 360 mg, or 480 mg flat dose), nivolumab injection can be infused undiluted or diluted so as not to exceed a total infusion volume of 160 mL. For patients weighing less than 40 kilograms (kg), the total volume of infusion must not exceed 4 mL per kg of patient weight.

During drug product preparation and handling, vigorous mixing or shaking is to be avoided. Instructions for dilution and infusion of nivolumab injection may be provided in the clinical protocol, pharmacy binder, pharmacy manual, or pharmacy reference sheet. Care must be taken to assure sterility of the prepared solution as the product does not contain any antimicrobial preservative or bacteriostatic agent. Nivolumab infusions are compatible with polyvinyl chloride (PVC) or polyolefin containers and infusion sets, and glass bottles.

Nivolumab Injection

Vials of nivolumab injection must be stored at 2^0C to 8^0C (36^0F to 46^0F) and protected from light and freezing. The unopened vials can be stored at room temperature (up to 25^0C, 77^0F) and room light for up to 48 hours. Undiluted Nivolumab Injection and Diluted Nivolumab Injection in the IV Container The administration of nivolumab infusion must be completed within 24 hours of preparation. If not used immediately, the infusion solution may be stored under refrigeration conditions (2°C to 8°C, 36°F to 46°F) for up to 24 hours, and a maximum of 8 hours of the total 24 hours can be at room temperature (up to 25□C, 77□F) and room light. The maximum of 8 hours under room temperature and room light conditions includes the product administration period.

7.2.2 **5-Fluorouracil (5-FU)**

7.2.2.1 Description

5-FU is an anti-metabolite of the pyrimidine analog type. Fluorouracil is considered to be cell cycle–specific for the S phase of cell division. Activity results from its conversion to an active metabolite in the tissues, and includes inhibition of DNA and RNA synthesis.

7.2.2.2 Mechanism of Action

Both normal and tumor cells metabolize 5-FU to 5-fluoro-2'-deoxyuridine monophosphate (FdUMP) and 5-fluorouridine triphosphate (FUTP). These metabolites cause cell injury by two different mechanisms. First, FdUMP and the folate cofactor, N 5-10 -methylenetetrahydrofolate, bind to thymidylate synthase (TS) to form a covalently bound ternary complex. This binding inhibits the formation of thymidylate from 2'-deoxyuridylate. Thymidylate is the necessary precursor of thymidine triphosphate, which is essential for the synthesis of DNA, so that a deficiency of this compound can inhibit cell division. Second, nuclear transcriptional enzymes can mistakenly incorporate FUTP in place of uridine triphosphate (UTP) during the synthesis of RNA. This metabolic error can interfere with RNA processing and protein synthesis.

7.2.2.3 Preparation and Administration

5-FU can be given as an I.V. bolus (slow push) or short (5-15 minutes) bolus infusion, or as a continuous infusion. Warm to body temperature before using. Forty six-forty eight-hour infusion of 5-FU should be prepared for administration via ambulatory infusion pump according to well-established standards. These solutions may be prepared in D5W or 0.9% NaCl.

7.2.2.4 How Supplied

Fluorouracil is available in 50 mg/mL (10 mL, 20 mL, 50 mL, 100 mL) vials. 500 mg was diluted in 5% dextrose to a total volume of 50 mL in a plasticized polyvinylchloride container (MVP drug reservoir). Storage without protection from light at room temperature and at 5 degrees C was continued for 4 months with periodic sampling. The stability of aqueous fluorouracil 50 mg/mL was also studied in portable infusion pumps under simulated infusion conditions. The drug was found to be stable for 7 days at 37 degrees C.

7.2.2.5 Storage Conditions

Fluorouracil solutions should be stored at 15 to 30 degrees Celsius (C) (59 to 86 degrees Fahrenheit (F) and protected from light. Solutions may discolor slightly but potency and safety are not adversely affected.

7.2.3 **Leucovorin**

7.2.3.1 Description

Leucovorin calcium is a reduced form of folic acid that stabilizes the binding of 5-dUMP and thymidylate synthetase, enhancing the activity of fluorouracil.

7.2.3.2 Human toxicology

When leucovorin and 5-fluorouracil (5-FU) are given concurrently, gastrointestinal toxicities, particularly stomatitis and diarrhea, are more common than those observed in patients treated with 5-FU alone.

7.2.3.3 Storage and Administration

Leucovorin calcium for injection should be stored at room temperature (25 degrees C; 77 degrees F). Leucovorin calcium for injection may be reconstituted with Bacteriostatic Water for Injection, USP,

which contains benzyl alcohol, and must be used within 7 days. It may also be reconstituted with Sterile Water for Injection, USP, and used immediately.

7.2.4 **Oxaliplatin**

7.2.4.1 Mechanism of Action

The main mechanism of action of oxaliplatin, like cisplatin, is mediated through the formation of DNA-adducts but DACH-platinum adducts are bulkier and more hydrophobic than cisplatin adducts. Oxaliplatin as a single agent has a broad spectrum of *in vitro* cytotoxic/antiproliferative activity against a variety of murine and human tumor cell lines. Oxaliplatin was also effective in cell lines with acquired cisplatin resistance.

7.2.4.2 Main Side Effects (**≥10%)**

Central nervous system: Peripheral neuropathy (may be dose limiting; 76% to 92%; acute 65%; grades 3/4: 5%; persistent 43%; grades 3/4: 3%), fatigue (61%), pain (14%), headache (13%), insomnia (11%)
Gastrointestinal: Nausea (64%), diarrhea (46%), vomiting (37%), abdominal pain (31%), constipation (31%), anorexia (20%), stomatitis (14%)
Hematologic: Anemia (64%; grades 3/4: 1%), thrombocytopenia (30%; grades 3/4: 3%), leukopenia (13%)
Hepatic: Increased serum AST (54%; grades 3/4: 4%), increased serum ALT (36%; grades 3/4: 1%), increased serum bilirubin (13%; grades 3/4: 5%)
Neuromuscular & skeletal: Back pain (11%)
Respiratory: Dyspnea (13%), cough (11%)
Miscellaneous: Fever (25%)

Less Frequent Side Effects (1% to 10%):
Cardiovascular: Edema (10%), chest pain (5%), peripheral edema (5%), flushing (3%), thromboembolism (2%)
Central nervous system: Rigors (9%), dizziness (7%)
Dermatologic: Skin rash (5%), alopecia (3%), palmar-plantar erythrodysesthesia (1%)
Endocrine & metabolic: Dehydration (5%), hypokalemia (3%)
Gastrointestinal: Dyspepsia (7%), dysgeusia (5%), flatulence (3%), hiccups (2%), mucositis (2%), dysphagia (acute 1% to 2%), gastroesophageal reflux disease (1%)
Genitourinary: Dysuria (1%)
Hematologic & oncologic: Neutropenia (7%)
Hypersensitivity: Hypersensitivity reaction (3%; includes urticaria, pruritus, facial flushing, shortness of breath, bronchospasm, diaphoresis, hypotension, syncope: grades 3/4: 2% to 3%)
Local: Injection site reaction (9%; redness, swelling, pain)
Neuromuscular & skeletal: Arthralgia (7%)
Ocular: Abnormal lacrimation (1%)
Renal: Increased serum creatinine (5% to 10%)
Respiratory: Upper respiratory tract infection (7%), rhinitis (6%), epistaxis (2%), pharyngitis (2%), pharyngolaryngeal dysesthesia (grades 3/4: 1% to 2%)

7.2.4.3 Preparation and Administration Precautions

Do not freeze and protect from light the concentrated solution. The solution must be further diluted in an infusion solution of 250-500 mL of 5% Dextrose Injection, USP. After final dilution with 250-500 mL of

5% Dextrose Injection, USP, the shelf life is 6 hours at room temperature (20-25°C [68-77°F]) or up to 24 hours under refrigeration (2-8°C [36-46°F]). After final dilution, protection from light is not required. Oxaliplatin is incompatible in solution with alkaline medications or media (such as basic solutions of 5-FU) and must not be mixed with these or administered simultaneously through the same infusion line. The infusion line should be flushed with D5W prior to administration of any concomitant medication.

7.2.4.4 How Supplied
Oxaliplatin is supplied in clear, glass, single-use vials with gray elastomeric stoppers and aluminum flip-off seals containing 50 mg or 100 mg of oxaliplatin as a sterile, preservative-free, aqueous solution at a concentration of 5 mg/mL. Water for Injection, USP is present as an inactive ingredient.

7.2.5 Irinotecan
7.2.51. Mechanism of Action:
Irinotecan and it's active metabolite, SN 38 are part of the class of Topoisomerase inhibitors which are drugs that interfere with the action of topoisomerase enzymes (topoisomerase I and II). Topoisomerase enzymes control the manipulation of the structure of DNA necessary for replication

7.2.5.2 Main Side Effects
Most common side effects: (≥10%)
Cardiovascular: Vasodilatation (9% to 11%)
Central nervous system: Cholinergic syndrome (47%; includes diaphoresis, flushing, increased peristalsis, lacrimation, miosis, rhinitis, sialorrhea), pain (23% to 24%), dizziness (15% to 21%), insomnia (19%), headache (17%), chills (14%)
Dermatologic: Alopecia (46% to 72%), diaphoresis (16%), skin rash (13% to 14%),
Endocrine & metabolic: Weight loss (30%), dehydration (15%)
Gastrointestinal: Diarrhea (late: 83% to 88%, grades 3/4: 14% to 31%; early: 43% to 51%, grades 3/4: 7% to 22%), nausea (70% to 86%), abdominal pain (57% to 68%), vomiting (62% to 67%), abdominal cramps (57%), anorexia (44% to 55%), constipation (30% to 32%), mucositis (30%), flatulence (12%), stomatitis (12%)
Hematologic: Anemia (60% to 97%; grades 3/4: 5% to 7%), leukopenia (63% to 96%, grades 3/4: 14% to 28%), thrombocytopenia (96%, grades 3/4: 1% to 4%), neutropenia (30% to 96%; grades 3/4: 14% to 31%)
Hepatic: Increased serum bilirubin (84%), increased serum alkaline phosphatase (13%)
Infection: Infection (14%)
Neuromuscular & skeletal: Weakness (69% to 76%), back pain (14%)
Respiratory: Dyspnea (22%), cough (17% to 20%), rhinitis (16%)
Miscellaneous: Fever (44% to 45%)

Less Frequent side effects (1% to 10%):
Cardiovascular: Edema (10%), hypotension (6%), thromboembolism (5%)
Central nervous system: Drowsiness (9%), confusion (3%)
Gastrointestinal: Abdominal distention (10%), dyspepsia (10%)
Hematologic: Febrile neutropenia (grades 3/4: 2% to 6%), hemorrhage (grades 3/4: 1% to 5%), neutropenic infection (grades 3/4: 1% to 2%)
Hepatic: Increased serum AST (10%), ascites (grades 3/4: ≤9%), jaundice (grades 3/4: ≤9%)
Respiratory: Pneumonia (4%)

7.2.5.3 Preparation and Administration Precautions
Administration: IV: Administer by IV infusion, usually over 90 minutes. Irinotecan injection must be diluted prior to infusion. Irinotecan should be diluted in 5% Dextrose Injection, USP, (preferred) or 0.9% Sodium Chloride Injection, USP, to a final concentration range of 0.12 to 2.8 mg/mL. In most clinical trials, Irinotecan isadministered in 250 mL to 500 mL of 5% Dextrose Injection, USP.

7.2.5.4 How Supplied
Irinotecan/Irinotecan Hydrochloride is supplied as an intravenous Injectable Solution: 1mL, 20mg

7.2.2 Dose Modifications for chemotherapy mFFX

FOLFIRINOX is a standard therapy for pancreatic cancers, therefore sites may use their discretion for dosing these regimens based on a participant's tolerability. Any laboratory-only abnormalities without clinical manifestations or electrolyte abnormalities that may be managed with supplementation also do not automatically need dose modification.

Doses will be reduced for hematologic and other non-hematologic toxicities. Dose adjustments are to be made according to the system showing the greatest degree of toxicity. Toxicities will be graded using the NCI CTCAE Version 4.3. If recurrent toxicities require dose modifications below those listed in Tables 1 and 2 below, the patient should be removed from study treatment. If patients experience study drug-related toxicities that require all treatment to be held for ≥ 14 days from their scheduled dose, they will be discontinued from further participation in this study. In special circumstances noted below, one component of study treatment may be permanently discontinued but the patient may remain on study. When the dose of one or more drugs is reduced due to toxicities, dose re-escalation will not be permitted for the duration of study treatment.

Table 1. Dose Reductions for FOLFIRINOX

	**Oxaliplatin**	**Irinotecan**	**5-FU**
**Starting dose level**	85 mg/m^2	150 mg/m^2	2400 mg/m^2
**Dose level -1**	65 mg/m^2	120 mg/m^2	1800 mg/m^2
**Dose level -2**	45 mg/m^2	90 mg/m^2	1200 mg/m^2

Table 2. Dose Modifications for Hematologic Toxicities at the Start of Each Cycle or Within a Cycle

Cycle Day	ANC (cells/mm^3)		Platelet count (cells/mm^3)	FFX
Day 1	<1500	OR	<100,000	Delay doses until recovery
Day 15	500 to <1000	OR	50,000 to <75,000	Withhold all chemo; resume at one dose level lower once counts adequately recover. Continue Nivolumab at same dose. Consider growth factor support.
	< 500	OR	<50,000	Withhold all doses (nivolumab) Resume all chemotherapy at one dose level lower once counts

				adequately recover. Consider growth factor support.

Abbreviations: ANC = Absolute Neutrophil Count

Table 3. Dose Modifications for Non-Hematologic Toxicity

For grade 3 or higher clinically relevant non-hematologic toxicities, treatment cycles will be delayed. Therapy may be resumed when the toxicity improves to grade 0-1 (or baseline). If the patients are unable to resume therapy within 2 weeks of a planned dose, the will be removed from the study. Dose adjustments in subsequent cycles will be made as follows depending on the highest grade of toxicity observed in prior cycles.

	**Irinotecan**	**Nivolumab**	**5-FU**	**Oxaliplatin**
**Diarrhea (Colitis)**				
Grade 1 -2	Continue at same dose			
Grade 3	Interrupt until grade 0-1<br>1st = Resume at decrease dose to level-1	Interrupt until grade 0-1<br>1st = Resume at same dose<br>2nd = Decrease dose to level-1	Interrupt until grade 0-1<br>1st = Resume at decrease dose to level-1	Interrupt until grade 0-1<br>1st = Resume at decrease dose to level-1
Grade 4	Interrupt until grade 0-1<br>1st = Decrease dose to level-2	Removal of agent	Interrupt until grade 0-1<br>1st = Decrease dose to level-2	Interrupt until grade 0-1<br>1st = Decrease dose to level-2
Symptomatic management of diarrhea: IV hydration and loperamide. Loperamide can be used at 4 mg at the first onset of loose stools and then 2 mg every 2 hours until the patient is diarrhea-free for at least 12 hours. If control takes longer than 2 days, medical evaluation including relevant diagnostic procedures and alternative treatments should be considered.				
**Nausea/Vomiting**				
Grade 1-2	Continue at same dose			
Grade 3	Interrupt until grade 0-1<br>1st = Decrease dose to level-1	Interrupt until grade 0-1<br>1st = Resume at same dose<br>2nd =	Continue at same dose	Interrupt until grade 0-1<br>1st = Decrease dose to level-1<br>2nd = Decrease dose to level-2

	**Irinotecan**	**Nivolumab**	**5-FU**	**Oxaliplatin**
	2nd = Decrease dose to level-2	Decrease dose to level-1		
Grade 4	Interrupt until grade 0-1<br>1st = Decrease dose to level-1<br>2nd = Discontinue study treatment	Interrupt until grade 0-1<br>1st = Decrease dose to level-1<br>2nd = Discontinue study treatment	Interrupt until grade 0-1<br>1st = Decrease dose to level-1<br>2nd = Discontinue study treatment	Interrupt until grade 0-1<br>1st = Decrease dose to level-1<br>2nd = Discontinue study treatment
**Liver Function Tests (AST, ALT, total bilirubin, NOT alkaline phosphatase)**				
Grade 1-2	Continue at same dose			
Grade 3	Interrupt until grade 0-1<br>1st = Decrease dose to level-1<br>2nd = Resume at same dose	Interrupt until grade 0-1<br>1st = Resume at same dose<br>2nd = Decrease dose to level-1	Continue at same dose	Interrupt until grade 0-1<br>1st = Decrease dose to level-1<br>2nd = Resume at same dose
Grade 4	Interrupt until grade 0-1<br>1st = Decrease dose to level-1<br>2nd = Off study	Discontinue study treatment	Interrupt until grade 0-1<br>1st = Decrease dose to level-1<br>2nd = Off study	Interrupt until grade 0-1<br>1st = Decrease dose to level-1<br>2nd = Off study
**Peripheral neuropathy**<br>Note: As observed in other clinical trials, ≥ Grade 3 neuropathy related to oxaliplatin is usually seen in later phases of the treatment (cycle 6 and beyond). If ≥ Grade 3 neuropathy occurs in early treatment cycles, other factors predisposing the patient to neuropathy might be present (e.g., Diabetes, alcohol consumption, concomitant medications). To maintain dose intensity during the				

	**Irinotecan**	**Nivolumab**	**5-FU**	**Oxaliplatin**
first 4 treatment cycles, careful consideration should be exercised when these predisposing factors are present. Oxaliplatin				
Grade 1-2	Continue at same dose	Continue at same dose	Continue at same dose	Continue at same dose
Grade 3	Continue at same dose		Continue at same dose. May give additional dexamethasone, muscle relaxer, or pain medication.	Dose reduce to next dose level, 65 mg/m^2
**Cutaneous toxicity**				
Grade 1	Continue at same dose.		Continue at same dose.	Continue at same dose
Grade 2-3	Continue treatment but reduce to next lower dose level; discontinue treatment if toxicity persists		Continue at same dose.	Continue at same dose
**Endocrinopathy Management Guidelines**				

**Asymptomatic TSH and glucose elevations**	**Continue Nivolumab per prototocol, include TSH, Hgb A1C at subsequent visits**	
**Symptomatic Endocrinopathy (Thyroid, diabetes, adrenal)**	**Evaluate with endocrinology consultation**<br><br>**Hold Nivolumab**	

			Irinotecan	Nivoluma b	5-FU	Oxaliplatin
**Suspicion of Adrenal Crisis**	**Hold Nivolumab**<br><br>**Stress dose steroids**					

GI Adverse Event Management Algorithm

Rule out non-inflammatory causes. If non-inflammatory cause is identified, treat accordingly and continue I-O therapy. Opiates/narcotics may mask symptoms of perforation. Infliximab should not be used in cases of perforation or sepsis.

Grade of Diarrhea/ Colitis

(NCI CTCAE v4)

Management

Grade 1

Diarrhea: < 4 stools/day over baseline;
Colitis: asymptomatic

- Continue I-O therapy per protocol

- Close monitoring for worsening symptoms.
- Educate patient to report worsening immediately
- Treat as Grade 2 or 3/4

Follow-up

Grade 2

Diarrhea: 4-6 stools per day over baseline; IV fluids indicated <24 hrs; not interfering with ADL

Colitis: abdominal pain; blood in stool

- Delay I-O therapy per protocol
- Symptomatic treatment

If improves to grade 1:
- Resume I-O therapy per protocol

If persists > 5-7 days or recur:
- 0.5-1.0 mg/kg/day methylprednisolone or oral equivalent
- When symptoms improve to grade 1, taper steroids over at least 1 month, consider prophylactic antibiotics for opportunistic infections, and resume I-O therapy per protocol.

If worsens or persists > 3-5 days with oral steroids:
- Treat as grade 3/4

Grade 3-4

Diarrhea (G3): ≥7 stools per day over baseline; incontinence; IV fluids ≥24 hrs; interfering with ADL

Colitis (G3): severe abdominal pain, medical intervention indicated, peritoneal signs

G4: life-threatening, perforation

- Discontinue I-O therapy per protocol
- 1.0 to 2.0 mg/kg/day methylprednisolone IV or IV equivalent
- Add prophylactic antibiotics for opportunistic infections
- Consider lower endoscopy

If improves:

- Continue steroids until grade 1, then taper over at least 1 month

If persists > 3-5 days, or recurs after improvement: Add infliximab 5 mg/kg (if no contraindication). Note: Infliximab should not be used in cases of perforation or sepsis

Patients on IV steroids may be switched to an equivalent dose of oral corticosteroids (e.g. prednisone) at start of tapering or earlier, once sustained clinical improvement is observed. Lower bioavailability of oral corticosteroids should be taken into account when switching to the equivalent dose of oral corticosteroids.

1 Renal Adverse Event Management Algorithm

Rule out non-inflammatory causes. If non-inflammatory cause, treat accordingly and continue I-O therapy.

Grade of Creatinine Elevation (NCI CTCAE v4)	Management	Follow-up
**Grade 1** Creatinine > ULN and > than baseline but ≤ 1.5x baseline	• Continue I-O therapy per protocol<br>• Monitor creatinine weekly	If returns to baseline:<br>•Resume routine creatinine monitoring per protocol<br>If worsens:<br>•Treat as Grade 2 or 3/4
**Grade 2-3**<br>Creatinine > 1.5x baseline to ≤ 6x ULN	• Delay I-O therapy per protocol<br>• Monitor creatinine every 2-3 days<br>• 0.5 to 1.0 mg/kg/day methylprednisolone IV or oral equivalent	If returns to Grade 1:<br>•Taper steroids over at least 1 month, consider prophylactic antibiotics for opportunistic infections, and resume I-O therapy and routine creatinine monitoring per protocol

Grade 4

Creatinine > 6x ULN

- Discontinue I-O therapy per protocol
- Monitor creatinine daily
- 1.0-2.0 mg/kg/day methylprednisolone IV or IV equivalent
- Consult nephrologist
- Consider renal biopsy

<u>If returns to Grade 1</u> : Taper steroids over at least 1 month and add prophylactic antibiotics for opportunistic infections

Patients on IV steroids may be switched to an equivalent dose of oral corticosteroids (e.g. prednisone) at start of tapering or earlier, once sustained clinical improvement is observed. Lower bioavailability of oral corticosteroids should be taken into account when switching to the equivalent dose of oral corticosteroids.

2 Pulmonary Adverse Event Management Algorithm

Rule out non-inflammatory causes. If non-inflammatory cause, treat accordingly and continue I-O therapy. Evaluate with imaging and pulmonary consultation.

Grade of Pneumonitis (NCI CTCAE v4)	Management	Follow-up
**Grade 1**<br>Radiographic changes only	• Consider delay of I-O therapy<br>• Monitor for symptoms every 2-3 days<br>• Consider Pulmonary and ID consults	• Re-image at least every 3 weeks<br>If worsens:
**Grade 2**<br>Mild to moderate new symptoms	• Delay I-O therapy per protocol<br>• Pulmonary and ID consults<br>• Monitor symptoms daily, consider hospitalization<br>• 1.0 mg/kg/day methyl-prednisolone IV or oral equivalent<br>• Consider bronchoscopy , lung biopsy	• Re-image every 1-3 days<br>If improves:<br>• When symptoms return to near baseline, tape at least 1 month and then resume I-O therapy and consider prophylactic antibiotics<br>If not improving after 2 weeks or worsening:<br>• Treat as Grade 3-4
**Grade 3-4**<br>Severe new symptoms; New/worsening hypoxia;	• Discontinue I-O therapy per protocol<br>• Hospitalize<br>• Pulmonary and ID consults<br>• 2-4 mg/kg/day methylprednisolone IV or IV equivalent<br>• [illegible]	If improves to baseline:<br>• Taper steroids over at least 6 weeks<br>If not improving after 48 hours or worsening:

Patients on IV steroids may be switched to an equivalent dose of oral corticosteroids (e.g. prednisone) at start of tapering or earlier, once sustained clinical improvement is observed. Lower bioavailability of oral corticosteroids should be taken into account when switching to the equivalent dose of oral corticosteroids

3 Hepatic Adverse Event Management Algorithm

Rule out non-inflammatory causes. If non-inflammatory cause, treat accordingly and continue I-O therapy. Consider imaging for obstruction.

Grade of Liver Test Elevation (NCI CTCAE v4)	Management	Follow-up
**Grade 1** AST or ALT > ULN to 3.0 x ULN and/or T. bili > ULN	• Continue I-O therapy per protocol	• Continue LFT monitoring per protocol<br>If worsens:
**Grade 2**<br>AST or ALT > 3.0 to ≤ 5 x ULN and/or T. bili > 1.5	• Delay I-O therapy per protocol<br>• Increase frequency of monitoring to every 3 days	If returns to baseline:<br>If elevations persist > 5-7 days or worsen :<br>• 0.5-1 mg/kg/day methylprednisolone or oral equivalent and when LFT returns to grade 1 or baseline, taper steroids over at least 1 month, consider prophylactic antibiotics for
**Grade 3-4**<br>AST or ALT > 5 x ULN or T.bili >3 x ULN	• Discontinue I-O therapy*<br>• Increase frequency of monitoring to every 1-2 days<br>• 1.0 to 2.0 mg/kg/day methylprednisolone IV or IV equivalent*<br>• Add prophylactic antibiotics for opportunistic infections<br>• Consult gastroenterologist	If returns to grade 2:<br>Taper steroids over at least 1 month<br>•<br>•

Patients on IV steroids may be switched to an equivalent dose of oral corticosteroids (e.g. prednisone) at start of tapering or earlier, once sustained clinical improvement is observed. Lower bioavailability of oral corticosteroids should be taken into account when switching to the equivalent dose of oral corticosteroids.

*The recommended starting dose for grade 4 hepatitis is 2 mg/kg/day methylprednisolone IV.

4 Endocrinopathy Adverse Event Management Algorithm

Rule out non-inflammatory causes. If non-inflammatory cause, treat accordingly and continue I-O therapy. Consider visual field testing, endocrinology consultation, and imaging.

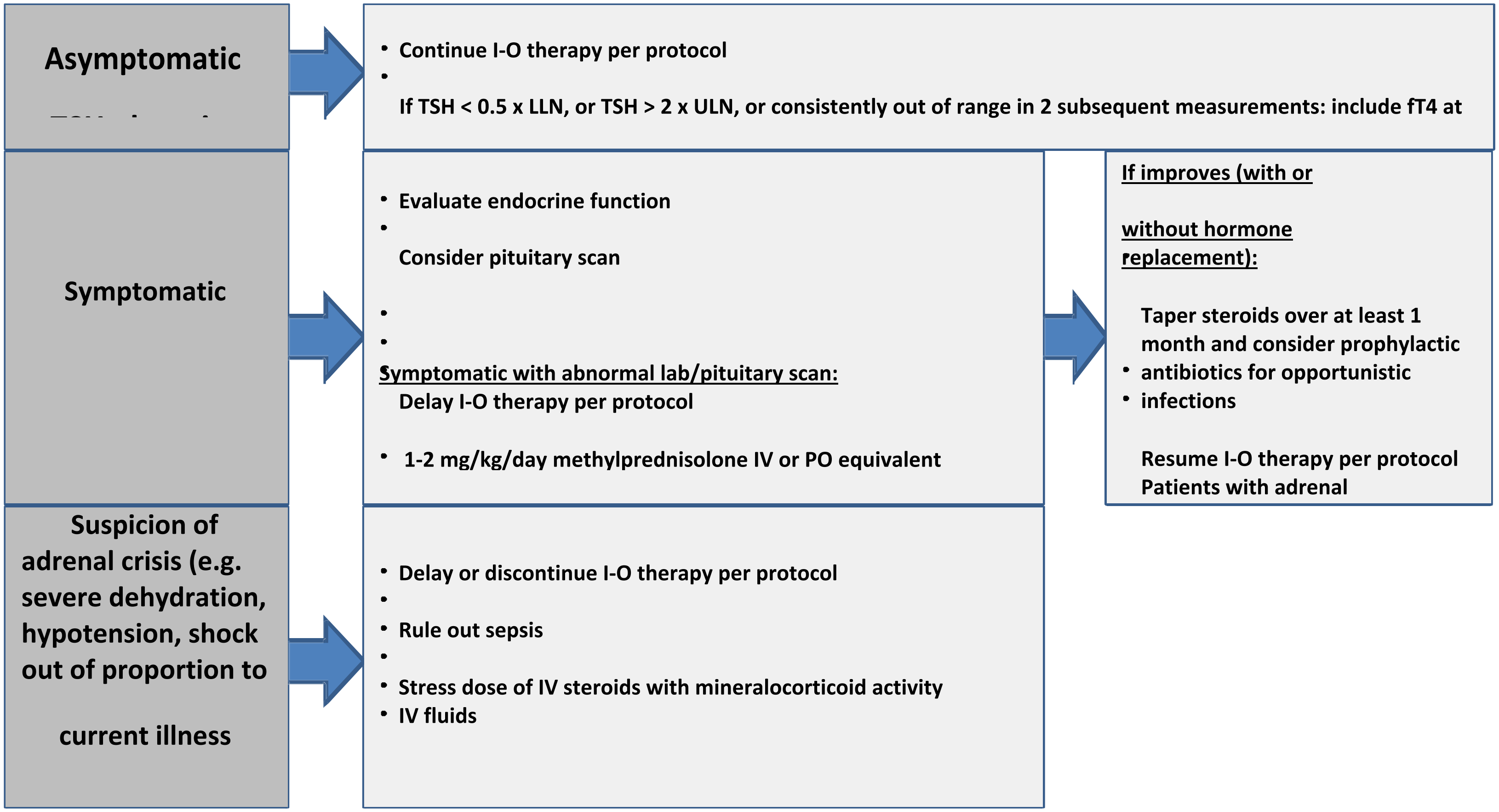

Patients on IV steroids may be switched to an equivalent dose of oral corticosteroids (e.g. prednisone) at start of tapering or earlier, once sustained clinical improvement is observed. Lower bioavailability of oral corticosteroids should be taken into account when switching to the equivalent dose of oral corticosteroids.

5 Skin Adverse Event Management Algorithm

Rule out non-inflammatory causes. If non-inflammatory cause, treat accordingly and continue I-O therapy.

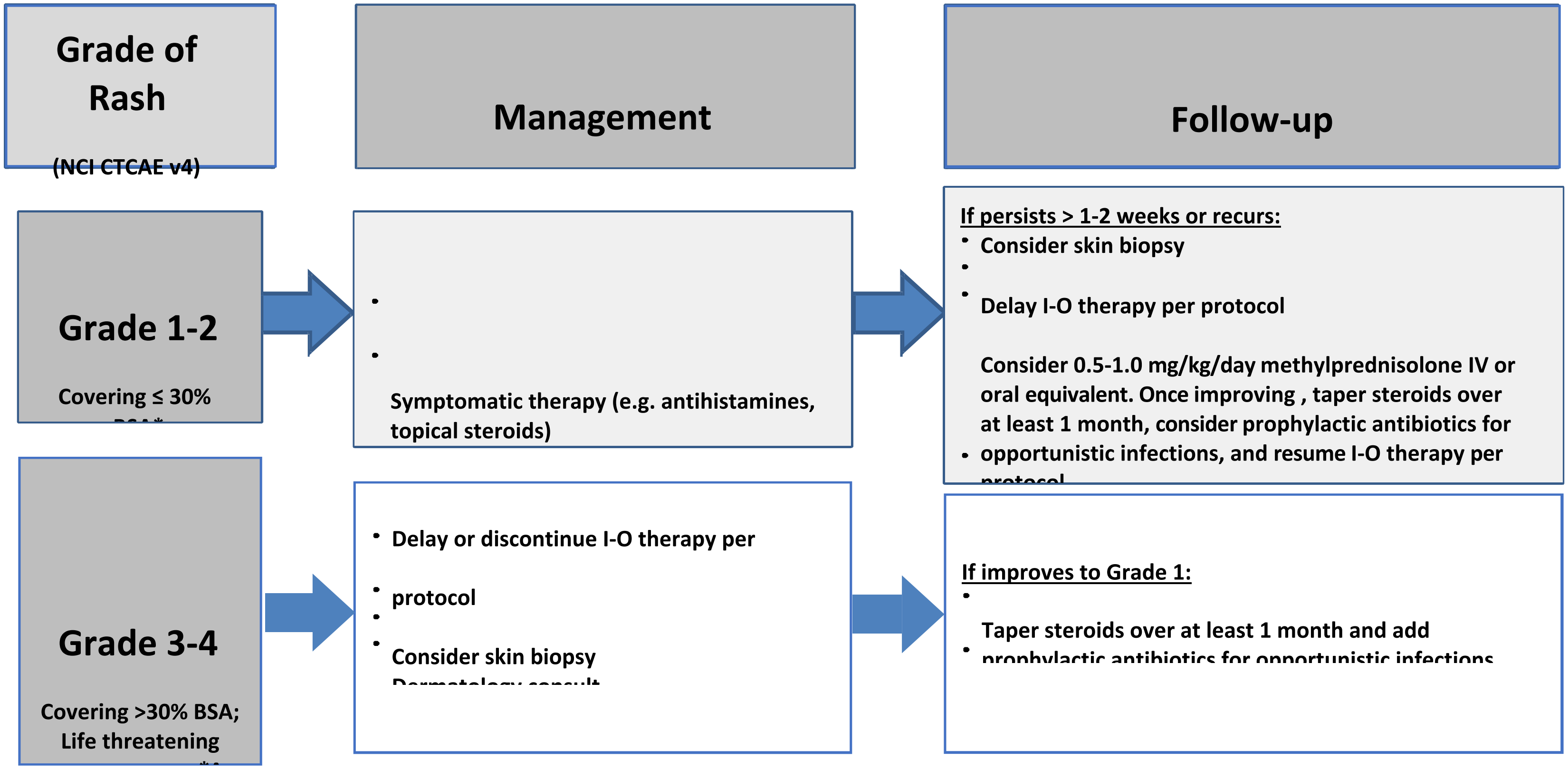

Patients on IV steroids may be switched to an equivalent dose of oral corticosteroids (e.g. prednisone) at start of tapering or earlier, once sustained clinical improvement is observed. Lower bioavailability of oral corticosteroids should be taken into account when switching to the equivalent dose of oral corticosteroids.

*Refer to NCI CTCAE v4 for term-specific grading criteria.

^If SJS/TEN is suspected, withhold I-O therapy and refer patient for specialized care for assessment and treatment. If SJS or TEN is diagnosed, permanently discontinue I-O therapy.

Neurological Adverse Event Management Algorithm

Rule out non-inflammatory causes. If non-inflammatory cause, treat accordingly and continue I-O therapy.

Grade of Neurological Toxicity (NCI CTCAE v4)	Management	Follow-up
**Grade 1**<br>Asymptomatic or mild symptoms; Intervention not [illegible]	Continue I-O therapy per protocol<br>•	Continue to monitor the patient.<br>If worsens:<br>Treat as Grade 2 or 3-4
**Grade 2**<br>Moderate symptoms; Limiting instrumental ADL	• Delay I-O therapy per protocol<br>• Treat symptoms per local guidelines<br>• Consider 0.5 to 1.0 mg/kg/day methylprednisolone IV or PO equivalent	If improves to baseline:<br>• Resume I-O therapy per protocol when improved to baseline<br>If worsens:<br>Treat as Grade 3-4
**Grade 3-4**<br>Severe symptoms; Limiting self-care ADL; Life-threatening	• Discontinue I-O therapy per protocol<br>• Obtain neurology consult<br>•<br>• Treat symptoms per local guidelines<br>1.0-2.0 mg/kg/day IV<br>•<br>methylprednisolone IV or IV equivalent<br>Add prophylactic antibiotics for opportunistic infections	If improves to Grade 2:<br>Taper steroids over at least 1 month<br>•

Patients on IV steroids may be switched to an equivalent dose of oral corticosteroids (e.g. prednisone) at start of tapering or earlier, once sustained clinical improvement is observed. Lower bioavailability of oral corticosteroids should be taken into account when switching to the equivalent dose of oral corticosteroids.

Toxicities of special note

- Nivolumab will be permanently discontinued for specific immune-mediated adverse events (as definied in the nivolumab investigator's brochure)

- Patients who develop Grade 3 or higher pulmonary toxicity will be taken off study.

- Patients who develop suspected Grade 3 or Grade 4 rash attributed to Nivolumab should be discontinued from nivolumab. This includes Steven's-Johnson Syndrome (SJS) or toxic epidermal necrolysis (TEN).

- Nivolumab should be discontinued if serum creatinine is >6 times the ULN.

- Nivolumab should be withheld for new-onset moderate or severe neurologic signs or symptoms and permanently discontinue for immune-mediated encephalitis

- Hypersensitivity reactions with nivolumab are rare. If they do occur, minor symptoms such as flushing, skin reactions, dyspnea, lower back pain, hypotension, or tachycardia may require temporary interruption of the infusion. However, severe reactions, such as hypotension requiring treatment, dyspnea requiring bronchodilators, angioedema or generalized urticaria require immediate discontinuation of study drug administration and aggressive symptomatic therapy. Patients who experience severe hypersensitivity reactions to nivolumab should not be re-challenged. They may continue on study with just mFFX.

- All treatment should be held for grade 3 or 4 febrile neutropenia, and not resumed until the fever has resolved and ANC ≥ 1500. All drugs within mFFX should then be resumed at the next lower dose level, while nivolumab may be resumed at the same dose level as previously.

- Patients who experience any other unexpected Grade 4 toxicities may be taken off study at the first or second episode, at the discretion of the investigator, per Table 4 above.

5.1 Study Drug Accountability

An accurate and current accounting of the dispensing and return of study drug for each subject will be maintained on an ongoing basis by a member of the study site staff. The study monitor will verify these documents throughout the course of the study.

6 STUDY PROCEDURES AND GUIDELINES

A Schedule of Events representing the required testing procedures to be performed for the duration of the study is diagrammed in Section 9.

Prior to conducting any study-related activities, written informed consent and the Health Insurance Portability and Accountability Act (HIPAA) authorization must be signed and dated by the subject

or subject's legal representative. If appropriate, assent must also be obtained prior to conducting any study-related activities.

6.1 Clinical Assessments

6.1.1 Concomitant Medications

All concomitant medication and concurrent therapies will be documented at screening/pre-treatment and on days 1 and 15 during the clinical assessment by the physician, and at early termination when applicable. Dose, route, unit frequency of administration, and indication for administration and dates of medication will be captured.

6.1.2 Demographics

Demographic information (date of birth, gender, race) will be recorded at screening.

6.1.3 Medical History

Relevant medical history, including history of current disease, other pertinent respiratory history, and information regarding underlying diseases will be recorded at screening.

6.1.4 Physical Examination

A complete physical examination will be performed by either the investigator or a subinvestigator who is a physician on day 1 of each cycle.

6.1.5 Vital Signs

Body temperature, blood pressure, pulse, oximetry and respirations will be performed after resting for 5 minutes on each day of therapy.

6.1.6 Adverse Events

Information regarding occurrence of adverse events will be captured throughout the study. Duration (start and stop dates), severity/grade, outcome, treatment and relation to study drug will be recorded on the case report form (CRF).

6.2 Clinical Laboratory Measurements

6.2.1 Hematology

Blood will be obtained and sent to clinical hematology lab for a complete blood count (hemoglobin, hematocrit, red blood cell count, white blood cell count, white blood cell differential, and platelet count). Hematology labs should be drawn within 2 days before each infusion.

6.2.2 Blood Chemistry Profile

Blood will be obtained and sent to clinical chemistry lab for determination of serum albumin, alkaline phosphatase, total bilirubin, bicarbonate, BUN, chloride, creatinine, glucose, potassium, SGOT [AST], SGPT [ALT], calcium, sodium. Blood chemistry profiles should be drawn within 2 days before Day 1 of each cycle.

6.2.3 Pregnancies

Pregnancies and suspected pregnancies (including a positive pregnancy test regardless of age or disease state) of a female subject occurring while the subject is on IP, or within (insert time-frame

which must be at least 28 days of the subject's last dose of IP), are considered immediately reportable events. IP is to be discontinued immediately.

The Investigator will follow the female subject until completion of the pregnancy. If the outcome of the pregnancy was abnormal (e.g., spontaneous or therapeutic abortion), the Investigator should report the abnormal outcome as an AE. If the abnormal outcome meets any of the serious criteria, it must be reported as an SAE.

- Women of childbearing potential (WOCBP) must agree to follow instructions for method(s) of contraception for the duration of study treatment with nivolumab and 5 months after the last dose of study treatment {i.e., 30 days (duration of ovulatory cycle) plus the time required for the investigational drug to undergo approximately five half-lives.}
- Males who are sexually active with WOCBP must agree to follow instructions for method(s) of contraception for the duration of study treatment with nivolumab and 7 months after the last dose of study treatment {i.e., 90 days (duration of sperm turnover) plus the time required for the investigational drug to undergo approximately five half-lives.}

All neonatal deaths that occur within 28 days of birth should be reported, without regard to causality, as SAEs.

Male Subjects
If a female partner of a male subject taking investigational product becomes pregnant, the male subject taking IP should notify the Investigator, and the pregnant female partner should be advised to call their healthcare provider immediately.

6.2.4 Pregnancy Test

A urine or serum pregnancy test will be obtained from female subjects who are of childbearing age prior to their participation in the study.

6.3 Tumor marker

CA19-9 levels will be measured prior to initiation of therapy and then monthly.

6.4 Research Tissue Analyses

EUS-guided core biopsies will be obtained at baseline, three core biopsies via 22-gauge "Pro-Core" needle will be obtained and will be submitted for research analyses purposes. Tissue will be placed in OCT in the EUS suite and will be picked up by the CC Tissue Bank for storage. Tissue analyses will include standard IHC staining for PDAC, but for CD3, CD8 and if limited tissue sample is obtained via the core biopsies, the priority of tissue analysis will be as follows: (1) PD1, PDL1, CD3, CD8, pan-immune cell CyTOF panel (32 antibodies for characterization of B lymphocytes, T lymphocytes, NK cells, MDSCs, iNKT, and gamma/delta T cells). (2) IFN-γ levels will be quantified and compared in fresh tissue lysates using an IFN-γ ELISA kit

7 EVALUATIONS BY VISIT

Patients will have baseline clinical and laboratory evaluations within 21 days prior to start of protocol therapy. Scans must be performed within 4 weeks prior to the day of the initiation of therapy. Patients' clinical assessment may take place on day 1 of each cycle. Laboratory

measurements should be done within 48 hours prior to the day of treatment with nivolumab and mFFX.

Table 4. Timeline of Clinical Assessment, Studies and Treatment

	Pre-Study	Chemotherapy Cycle (n=36)				Post-Surgery
		1	2	3	4-6 (if necessary)	
**Nivolumab**		X	X	X	X	
**mFFX**		X	X	X	X[l]	XX[m] (adjuvant therapy if successful resection)
**Informed Consent and Eligibility Review**	X					
**Demographics (date of birth, gender, race)**	X					
**Medical History**	X					
**Review of Concurrent Medications[a]**	X	X	X	X	X	X
**Physical Examination[b]**	X	X	X	X	X	X
**Vital Signs[b]**	X	X	X	X	X	
**Height**	X					
**Weight[a]**	X	X	X	X	X	X
**ECOG Performance Status[a]**	X	X	X	X	X	X
**CBC with Differential[c]**	X	X	X	X	X	X
**Blood Chemistry Profile[c]**	X	X	X	X	X	X
**CA 19-9**	X	X	X	X	X	X
**Thyroid Function (TSH, T4)**	X		X		X	X
**Hepatitis B serology (Hepatitis B core Antibody, Hepatitis B surface Antibody, Hepatitis B surface Antigen)**	X					
**Serum or urine β-hcG**	X	X	X	X	X	
**Adverse Event Evaluation[a]**	X	X	X	X	X	X
**CT Chest, Abdomen, Pelvis with Contrast[d]**			X		X (day 21)	
**EUS-guided Core Biopsy[d] (n=15)**	X					

a. These measurements will be obtained bi-weekly (draw within 2 days of infusion) while receiving chemotherapy.

b. Physical examination will occur on day 1 of each cycle of neoadjuvant chemotherapy (or more frequently at the treating provider's discretion) and be performed by the treating MD or qualified delegate

c. Complete metabolic panel:: Albumin, alkaline phosphatase, total bilirubin, bicarbonate, BUN, calcium, chloride, creatinine, glucose, potassium, SGOT [AST], SGPT [ALT], sodium.
d. The diagnostic CT scan or MRI confirming resectable or borderline resectability must have been performed within 4 weeks prior to the day of the intiation of therapy.

Surgery should be scheduled no earlier than 2 week and, ideally, no later than 4 weeks post-neoadjuvant therapy. Scheduling of surgery is at the discretion of the physician per the clinical condition of the patient.

m. Adjuvant chemotherapy should start no earlier than 8 weeks and no later than 12 weeks post-surgery. Choice of therapy will be made according to the treating MD's discretion.

8 ADVERSE EXPERIENCE REPORTING AND DOCUMENTATION

An adverse event (AE) is any untoward medical occurrence in a clinical investigation of a patient administered a pharmaceutical product and that does not necessarily have a causal relationship with the treatment. An AE is therefore any unfavorable and unintended sign (including an abnormal laboratory finding), symptom or disease temporally associated with the administration of an investigational product, whether or not related to that investigational product. An unexpected AE is one of a type not identified in nature, severity, or frequency in the current Investigator's Brochure or of greater severity or frequency than expected based on the information in the Investigator's Brochure.

The Investigator and the study team will record any occurrences of AEs reported during conversations with the patients or their family members or care providers.. This will occur from the signing of the informed consent to 28 days after the last dose of study drug, or longer if the PI deems the event is related to the drug. Adverse events will be recorded in the patient CRF. Adverse events will be described by duration (start and stop dates and times), severity, outcome, treatment and relation to study drug, or if unrelated, the cause.

AE Severity

The National Cancer Institute's Common Terminology Criteria for Adverse Events (CTCAE) Version 4.0 should be used to assess and grade AE severity, including laboratory abnormalities judged to be clinically significant. The modified criteria can be found in the study manual. If the experience is not covered in the modified criteria, the guidelines shown in Table 5 below should be used to grade severity. It should be pointed out that the term "severe" is a measure of intensity and that a severe AE is not necessarily serious.

IND Annual Reports

Since the FDA has granted an IND number, it is a requirement of 21 CFR 312.33, that an annual report is provided to the FDA within 60-days of the IND anniversary date. 21 CRF 312.33 provides the data elements that are to be submitted in the report. The Annual Report should be

filed in the study's Regulatory Binder, and a copy provided to BMS as a supporter of this study as follows.

Table 5. AE Severity Grading

Severity (Toxicity Grade)	Description
Mild (1)	Transient or mild discomfort; no limitation in activity; no medical intervention or therapy required. The subject may be aware of the sign or symptom but tolerates it reasonably well.
Moderate (2)	Mild to moderate limitation in activity, no or minimal medical intervention/therapy required.
Severe (3)	Marked limitation in activity, medical intervention/therapy required hospitalizations possible.
Life-threatening (4)	The subject is at risk of death due to the adverse experience as it occurred. This does not refer to an experience that hypothetically might have caused death if it were more severe.
Death related to adverse events (5)	While the subject is on study, death attributed to any cause will be reported.

AE Relationship to Study Drug

The relationship of an AE to the study drug should be assessed using the following the guidelines in NCI CTCAE v. 4.3.

Table 6. AE Relationship to Study Drug

Relationship to Drug	Comment
Definitely	Previously known toxicity of agent; or an event that follows a reasonable temporal sequence from administration of the drug; that follows a known or expected response pattern to the suspected drug; that is confirmed by stopping or reducing the dosage of the drug; and that is not explained by any other reasonable hypothesis.
Probably	An event that follows a reasonable temporal sequence from administration of the drug; that follows a known or expected response pattern to the suspected drug; that is confirmed by stopping or reducing the dosage of the drug; and that is unlikely to be explained by the known characteristics of the subject's clinical state or by other interventions.
Possibly	An event that follows a reasonable temporal sequence from administration of the drug; that follows a known or expected response pattern to that suspected drug; but that could readily have been produced by a number of other factors.
Unrelated	An event that can be determined with certainty to have no relationship to the study drug.

8.1 Serious Adverse Experiences (SAE)

A **S*erious Adverse Event (SAE)*** is any untoward medical occurrence that at any dose:

- results in death

- is life-threatening (defined as an event in which the participant was at risk of death at the time of the event; it does not refer to an event which hypothetically might have caused death if it were more severe)
- requires inpatient hospitalization or causes prolongation of existing hospitalization (see **NOTE** below)
- results in persistent or significant disability/incapacity
- is a congenital anomaly/birth defect
- is an important medical event (defined as a medical event(s) that may not be immediately life-threatening or result in death or hospitalization but, based upon appropriate medical and scientific judgment, may jeopardize the subject or may require intervention [eg, medical, surgical] to prevent one of the other serious outcomes listed in the definition above.) Examples of such events include, but are not limited to, intensive treatment in an emergency room or at home for allergic bronchospasm; blood dyscrasias or convulsions that do not result in hospitalization.)
- Suspected transmission of an infectious agent (eg, pathogenic or nonpathogenic) via the study drug is an SAE.

Although pregnancy, overdose, potential drug-induced liver injury (DILI), and cancer are not always serious by regulatory definition, these events must be handled as SAEs.

Any component of a study endpoint that is considered related to study therapy should be reported as an SAE (eg, death is an endpoint, if death occurred due to anaphylaxis, anaphylaxis must be reported).

The following hospitalizations are not considered SAEs:

- a visit to the emergency room or other hospital department < 24 hours, that does not result in admission (unless considered an important medical or life-threatening event)
- elective surgery, planned prior to signing consent
- admissions as per protocol for a planned medical/surgical procedure
- routine health assessment requiring admission for baseline/trending of health status (eg, routine colonoscopy)
- Medical/surgical admission other than to remedy ill health and planned prior to entry into the study. Appropriate documentation is required in these cases.
- Admission encountered for another life circumstance that carries no bearing on health status and requires no medical/surgical intervention (eg, lack of housing, economic inadequacy, caregiver respite, family circumstances, administrative reason).
- Admission for administration of anticancer therapy in the absence of any other SAEs (applies to oncology protocols)

8.1.1 Serious Adverse Experience Reporting

Study sites will document all SAEs that occur (whether or not related to study drug) per UCLACHR Guidelines. The collection period for all SAEs will begin after informed consent is obtained and end after procedures for the final study visit have been completed and at 100 days after the last dose of study drug or longer if the PI determines the event is related to the study drug.

In accordance with the standard operating procedures and policies of the local Institutional Review Board (IRB)/Independent Ethics Committee (IEC), the site investigator will report SAEs to the IRB/IEC.

Expedited Reporting
Reporting to the UCLA Data and Safety Monitoring Committee (DSMC)
If a death occurs during the treatment phase of the study or within 30 days after the last administration of the study drug(s) and it is determined to be related either to the study drug(s) or to a study procedure, the Investigator or his/her designee must notify the DSMC Chair (or qualified alternate) within 1 business day of knowledge of the event. The contact may be by phone or e-mail.

Reporting to UCLA's Institutional Review Board (IRB)
The Study Chair must report events meeting the CHR definition of "Unanticipated Problem" (UP) within 10 business days of his/her awareness of the event.

Guidance on Adverse Event Reporting to the CHR is available online at the UCLA Human Research Protection Program website.

Expedited Reporting to the Food and Drug Administration (FDA)
As this study is being conducted under an Investigational New Drug Application (IND), the Sponsor-Investigator is responsible for determining whether or not the suspected adverse reaction meets the criteria for expedited reporting in accordance with Federal Regulations (21 CFR §312.32).

The Investigator must report in an IND safety report any suspected adverse reaction that is both serious and unexpected. The Sponsor-Investigator needs to ensure that the event meets all three definitions:

- Suspected adverse reaction
- Serious
- Unexpected

If the adverse event does not meet all three of the definitions, it should not be submitted as an expedited IND safety report.

The timeframe for submitting an IND safety report to FDA is no later than **15 calendar days** after the Investigator determines that the suspected adverse reaction qualifies for reporting (21 CFR 312.32(c)(1)).

Any unexpected fatal or life-threatening suspected adverse reaction must be reported to FDA no later than **7 calendar days** after the Investigator's initial receipt of the information (21 CFR 312.32(c)(2)).

Any relevant additional information that pertains to a previously submitted IND safety report must be submitted to FDA as a Follow-up IND Safety Report without delay, as soon as the information is available (21 CFR 312.32(d)(2)).

The investigator (or designee) should prepare Form FDA 3500A (MedWatch) detailing the event, and contact the Institutional Trials Unit for assistance in the preparation of the IND Safety Report.

8.1.2 Reporting of Serious Adverse Events to BMS

To ensure patient safety, every SAE, regardless of suspected causality, occurring after the patient has provided informed consent and during the study period will be reported to BMS within 24 hours of learning of its occurrence. SAEs will be reported to BMS by completed SAE Form or MedWatch 3500A form via email at safety@BMS.com or fax at 609-818-3804. The form should include a description of the event, subject number/initials, criteria for seriousness, severity, assessment of relationship to study drug(s), whether the event was expected or unexpected per the Investigator's Brochure or package insert, and other available information relevant to the event. Any new information about a previously reported SAE must also be sent to BMS within 24 hours of the initial awareness. The investigator must also provide BMS with a copy (MedWatch 3500A form) of any FDA submissions (expedited 7-Day or 15-Day initial and follow-up safety reports) within 24 hours of submission.

- All Serious Adverse Events (SAEs) that occur following the subject's written consent to participate in the study through 100 days of discontinuation of dosing must be reported to BMS Worldwide Safety, whether related or not related to study drug. If applicable, SAEs must be collected that relate to any later protocol-specified procedure (eg, a follow-up skin biopsy).
- Following the subject's written consent to participate in the study, all SAEs, whether related or not related to study drug, are collected, including those thought to be associated with protocol-specified procedures. The investigator should report any SAE occurring after these aforementioned time periods, which is believed to be related to study drug or protocol-specified procedure.
- An SAE report should be completed for any event where doubt exists regarding its seriousness;
- If the investigator believes that an SAE is not related to study drug, but is potentially related to the conditions of the study (such as withdrawal of previous therapy or a complication of a study procedure), the relationship should be specified in the narrative section of the SAE Report Form.
- If the BMS safety address is not included in the protocol document (eg, multicenter studies where events are reported centrally), the procedure for safety reporting must be reviewed/approved by the BMS Protocol Manager. Procedures for such reporting must be reviewed and approved by BMS prior to study activation.

An appropriate SAE form (e.g. ex-US = CIOMS form or USA = Medwatch form) should be used to report SAEs to BMS. If you prefer to use your own Institutional form, it must be reviewed by BMS prior to study initiation. Note: Please include the BMS Protocol number on the SAE form or on the cover sheet with the SAE form transmission.

  - The CIOMS form is available at: http://www.cioms.ch/index.php/cioms-form-i
  - The MedWatch form is available at: MedWatch 3500 Form

-
- Worldwide.Safety@bms.comaepbusinessprocess@bms.com

- In accordance with local regulations, BMS will notify investigators of all reported SAEs that are suspected (related to the investigational product) and unexpected (ie, not previously described in the IB). An event meeting these criteria is termed a Suspected, Unexpected Serious Adverse Reaction (SUSAR). Investigator notification of these events will be in the form of a SUSAR Report.
  - Other important findings which may be reported by BMS as an Expedited Safety Report (ESR) include: increased frequency of a clinically significant expected SAE, an SAE considered associated with study procedures that could modify the conduct of the study, lack of efficacy that poses significant hazard to study subjects, clinically significant safety finding from a nonclinical (eg, animal) study, important safety recommendations from a study data monitoring committee, or sponsor decision to end or temporarily halt a clinical study for safety reasons.
  - Upon receiving an ESR from BMS, the investigator must review and retain the ESR with the IB. Where required by local regulations or when there is a central IRB/IEC for the study, the sponsor will submit the ESR to the appropriate IRB/IEC. The investigator and IRB/IEC will determine if the informed consent requires revision. The investigator should also comply with the IRB/IEC procedures for reporting any other safety information.
  - In addition to the Sponsor Investigator's responsibility to report events to their local HA, suspected serious adverse reactions (whether expected or unexpected) shall be reported by BMS to the relevant competent health authorities in all concerned countries according to local regulations (either as expedited and/or in aggregate reports).

SAEs, whether related or not related to study drug, and pregnancies must be reported to BMS within 24 hours \ 1 Business Day of becoming aware of the event. SAEs must be recorded on either CIOMS, MedWatch, or approved site SAEform.

Pregnancies must be reported and submitted to BMS on any of the following form(s):

1. MedWatch or, CIOMS or
2. BMS Pregnancy Surveillance Form or,
3. Approved site SAE form

ADVERSE EVENTS

An Adverse Event (AE) is defined as any new untoward medical occurrence or worsening of a preexisting medical condition in a clinical investigation participant administered study drug and that does not necessarily have a causal relationship with this treatment. An AE can therefore be any unfavorable and unintended sign (such as an abnormal laboratory finding), symptom, or disease temporally associated with the use of investigational product, whether or not considered related to the investigational product.

The causal relationship to study drug is determined by a physician and should be used to assess all adverse events (AE). The casual relationship can be one of the following:

Related: There is a reasonable causal relationship between study drug administration and the AE.
Not related: There is not a reasonable causal relationship between study drug administration and the AE.

The term "reasonable causal relationship" means there is evidence to suggest a causal relationship. Adverse events can be spontaneously reported or elicited during open-ended questioning, examination, or evaluation of a subject. (In order to prevent reporting bias, subjects should not be questioned regarding the specific occurrence of one or more AEs.)

NONSERIOUS ADVERSE EVENT

- Non-serious Adverse Events (AE) are to be provided to BMS in aggregate via interim or final study reports as specified in the agreement or, if a regulatory requirement [eg, IND US trial] as part of an annual reporting requirement.
- Non-serious AE information should also be collected from the start of a placebo lead-in period or other observational period intended to establish a baseline status for the subjects.

A ***non-serious adverse event*** is an AE not classified as serious.

Non-serious Adverse Event Collection and Reporting

The collection of non-serious AE information should begin at initiation of study drug. All non-serious adverse events (not only those deemed to be treatment-related) should be collected continuously during the treatment period and for a minimum of 100days following the last dose of study treatment.

Non-serious AEs should be followed to resolution or stabilization, or reported as SAEs if they become serious. Follow-up is also required for non-serious AEs that cause interruption or discontinuation of study drug and for those present at the end of study treatment as appropriate.

Laboratory Test Abnormalities

All laboratory test results captured as part of the study should be recorded following institutional procedures. Test results that constitute SAEs should be documented and reported to BMS as such. The following laboratory abnormalities should be documented and reported appropriately:

- any laboratory test result that is clinically significant or meets the definition of an SAE
- any laboratory abnormality that required the participant to have study drug discontinued or interrupted
- any laboratory abnormality that required the subject to receive specific corrective therapy.

It is expected that wherever possible, the clinical rather than laboratory term would be used by the reporting investigator (eg, anemia versus low hemoglobin value).

Potential Drug Induced Liver Injury (DILI)

Specific criteria for identifying potential DILI have not been identified for this protocol. Standard medical practice in identifying and monitoring hepatic issues should be followed.

Wherever possible, timely confirmation of initial liver-related laboratory abnormalities should occur prior to the reporting of a potential DILI event. All occurrences of potential DILIs, meeting the defined criteria, must be reported as SAEs.

Potential drug induced liver injury is defined as:

1) AT (ALT or AST) elevation > 3 times upper limit of normal (ULN)

AND

2) Total bilirubin > 2 times ULN, without initial findings of cholestasis (elevated serum alkaline phosphatase)

AND

3) No other immediately apparent possible causes of AT elevation and hyperbilirubinemia, including, but not limited to, viral hepatitis, pre-existing chronic or acute liver disease, or the administration of other drug(s) known to be hepatotoxic.

Wherever possible, timely confirmation of initial liver-related laboratory abnormalities should occur prior to the reporting of a potential DILI event. All occurrences of potential DILIs, meeting the defined criteria, must be reported as SAEs.

Pregnancy

If, following initiation of the investigational product, it is subsequently discovered that a study participant is pregnant or may have been pregnant at the time of investigational product exposure, including during at least 5 half-lives after product administration, the investigational product will be permanently discontinued in an appropriate manner (eg, dose tapering if necessary for participant).

The investigator must immediately notify Worldwide.Safety@bms.com of this event via either the CIOMS, MedWatch or appropriate Pregnancy Surveillance Form in accordance with SAE reporting procedures.

Protocol-required procedures for study discontinuation and follow-up must be performed on the participant.

Follow-up information regarding the course of the pregnancy, including perinatal and neonatal outcome and, where applicable, offspring information must be reported on the CIOMS, MedWatch, BMS Pregnancy Surveillance Form, **or** approved site SAE form. A BMS Pregnancy Surveillance Form may be provided upon request.

Any pregnancy that occurs in a female partner of a male study participant should be reported to BMS. Information on this pregnancy will be collected on the Pregnancy Surveillance Form. In order for Sponsor or designee to collect any pregnancy surveillance information from the female partner, the female partner must sign an informed consent form for disclosure of this information.

Overdose

An overdose is defined as the accidental or intentional administration of any dose of a product that is considered both excessive and medically important. All occurrences of overdose must be reported as an SAE.

Other Safety Considerations

Any significant worsening noted during interim or final physical examinations, electrocardiograms, X-rays, and any other potential safety assessments, whether or not these

procedures are required by the protocol, should also be recorded as a non-serious or serious AE, as appropriate, and reported accordingly.

8.2 Data and Safety Monitoring Committee (DSMC) Contacts

DSMC Chair:	Sven DeVos, MD
Phone:	310-633-8400
Email:	sdevos@mednet.ucla.edu
Address:	2338 PVUB<br>UCLA<br>Los Angeles, CA 90095

9 DISCONTINUATION AND REPLACEMENT OF SUBJECTS

9.1 Early Discontinuation of Study Drug

A subject may be discontinued from study treatment at any time if the subject or the investigator feels that it is not in the subject's best interest to continue. The following is a list of possible reasons for study treatment discontinuation:

- Subject withdrawal of consent (or assent)
- Subject is not compliant with study procedures
- Adverse event that in the opinion of the investigator would be in the best interest of the subject to discontinue study treatment. This includes unexpected delay in wound healing, development of wound dehiscence or pancreatic anastomotic leak.
- Protocol violation requiring discontinuation of study treatment
- Lost to follow-up
- Sponsor request for early termination of study
- Positive pregnancy test (females)
- Initiation of other anticancer therapy
- Development of intercurrent medical condition or need for concomitant treatment that precludes further safe participation in the trial such as the occurrence of any Grade thromboembolic event.

If a subject is withdrawn from treatment due to an adverse event, the subject will be followed and treated by the Investigator until the abnormal parameter or symptom has resolved or stabilized. All subjects who discontinue study treatment should come in for an early discontinuation visit as soon as possible and then should be encouraged to complete all remaining scheduled visits and procedures.

All subjects are free to withdraw from participation at any time, for any reason, specified or unspecified, and without prejudice.

Reasonable attempts will be made by the investigator to provide a reason for subject withdrawals. The reason for the subject's withdrawal from the study will be specified in the subject's source documents Refer to Section 9 for early termination procedures.

9.2 Withdrawal of Subjects from the Study

A subject may be withdrawn from the study at any time if the subject or the investigator feels that it is not in the subject's best interest to continue.

All subjects are free to withdraw from participation at any time, for any reason, specified or unspecified, and without prejudice.

Reasonable attempts will be made by the investigator to provide a reason for subject withdrawals. The reason for the subject's withdrawal from the study will be specified in the subject's source documents. As noted above, subjects who discontinue study treatment early (i.e., they withdraw prior to visit 2) should have an early discontinuation visit. Refer to Section 9 for early termination procedures. Subjects who withdraw after first cycle with study therapy but prior to second treatment cycle should be encouraged to come in for a final visit (and the procedures to be followed would include those for their next scheduled visit).

9.3 Replacement of Subjects

Patients will be included in the primary AE evaluation if the full 28-day treatment cycle is completed. In the event that a patient is unable to complete the 28-day cycle unrelated to treatment toxicity, this patient will be removed from study and will be replaced for primary MTD evaluation. In the event that a patient withdraws from study due to treatment-related toxicity, this patient will be included in the AE evaluation for this study and will not be replaced.

10 PROTOCOL VIOLATIONS

A protocol violation occurs when the subject or the investigator fails to adhere to significant protocol requirements affecting the inclusion, exclusion, subject safety and primary endpoint criteria. Protocol violations for this study include, but are not limited to, the following:

- Failure to meet inclusion/exclusion criteria
- Use of a prohibited concomitant medication

Failure to comply with Good Clinical Practice (GCP) guidelines will also result in a protocol violation. The investigator will determine if a protocol violation will result in withdrawal of a subject.

When a protocol violation occurs, it will be discussed with the investigator and a Protocol Violation Form detailing the violation will be generated. A copy of the form will be filed in the site's regulatory binder.

11 STATISTICAL METHODS AND CONSIDERATIONS

Prior to the analysis of the final study data, a detailed Statistical Analysis Plan (SAP) will be written describing all analyses that will be performed. The SAP will contain any modifications to the analysis plan described below.

11.1 Data Sets Analyzed

All eligible patients who are enrolled into the study and receive at least one complete, 28-day cycle of the study drugs will be included in the safety analysis. If the patient withdraws from the study prior to completing the first cycle of chemotherapy secondary to reasons other than toxicity related

to the study therapy, they will not be included in the primary analysis. However, if the patients' enrollment in the trial is terminated secondary to toxicity secondary to the study drugs, they will be evaluated as having an AE and will be included in the analyses.

In addition, the patients who have received at least 2 complete, 28-day cycles of study drugs will be included in the secondary analyses as they will have had monthly CA 19-9 measurements and restaging scans which would be obtained after 12 weeks of therapy.

11.2 Demographic and Baseline Characteristics

The following demographic variables at screening will be summarized: race, gender, age, height and weight.

11.3 Analysis of Primary Endpoint

The primary endpoint of the study is to evaluate development of clinically relevant pancreatic fistula in the post-operative period after neoadjuvant treatment with nivolumab and mFFX. The co-primary endpoint is to evaluate pathologic complete response after nivolumab and mFFX treatment. Descriptive statistics with frequency and proportion will be used to evaluate the primary endpoints.

11.4 Analysis of Secondary Endpoint

Secondary endpoints include early efficacy as measured by percent change of CA 19-9 response rate, R0 resection rate, overall response rate (ORR) and disease free survival (DFS). Descriptive statistics with frequency and proportion will be used to analyze the CA19-9 response rate and ORR. Kaplan-Meier methods will be used to analyze DFS with median and 95% CI.

11.5 Analyses of the Correlative Studies

Tissue Analyses:

We will examine the tissue from baseline to post-therapy at the time of surgery or at the time of progression. We will use descriptive statistics and graphical displays to compare the percent change in stromal depletion overall and to describe the association with cell proliferation and death. In addition, we will graphically explore the percent change in stromal depletion for patients who undergo surgery compared to those who have disease progression.

14.6 Additional Stopping Rules for Safety

In order to ensure patient safety, this trial includes early stopping rules in the event of unexpected postoperative complications such as development of pancreatic fistula. Two interim safety assessments will be performed when first 7 and 15 evaluable patients are enrolled in the study. If three out of first 7 patients or four out of first 15 patients show unexpected post-operative complications with delayed wound healing, development of wound dehiscence, wound infection or pancreatic fistula, this trial will be stopped. The early stopping rules of declaring the trial unsafe based on assumed postoperative complications of < 11% and type I alpha of 10% are described in Table 1.

Table 9. Early Stopping Criteria (assuming %complications < 11%)

**# of evaluable patients enrolled**	7	15

**Alpha (Type I error rate)**	10%	10%	5%
**# of post-op complications**	3	4	5

Furthermore, the development of venous thromboembolic events (DVT and/or PE) or arterial thromboembolic events during the preoperative treatment period may affect the safety and timing of surgery. Therefore, at the same interim assessment time points as above, we will consider the development of VTE/ATEs in 3 or more of the first 7 patients, or 4 or more of the first 15 patients, occurring during the preoperative treatment period through one week postoperatively, as a signal to stop the trial early.

12 DATA COLLECTION, RETENTION AND MONITORING

12.1 Data Collection Instruments

The Investigator will prepare and maintain adequate and accurate source documents designed to record all observations and other pertinent data for each subject treated with the study drug.
Study personnel will enter data from source documents corresponding to a subject's visit into the protocol-specific electronic Case Report Form (eCRF) OR paper CRF when the information corresponding to that visit is available. Subjects will not be identified by name in the study database or on any study documents to be collected by the study personnel, but will be identified by a site number, subject number and initials.

For eCRFs: If a correction is required for an eCRF, the time and date stamps track the person entering or updating eCRF data and creates an electronic audit trail. *For paper CRFs:* If a correction is made on a CRF, the study staff member will line through the incorrect data, write in the correct data and initial and date the change.

The Investigator is responsible for all information collected on subjects enrolled in this study. All data collected during the course of this study must be reviewed and verified for completeness and accuracy by the Investigator. A copy of the CRF will remain at the Investigator's site at the completion of the study.

12.2 Data Management Procedures

The data will be entered into a validated database. The Data Management group will be responsible for data processing, in accordance with procedural documentation. Database lock will occur once quality assurance procedures have been completed.
All procedures for the handling and analysis of data will be conducted using good computing practices meeting FDA guidelines for the handling and analysis of data for clinical trials.

12.3 Data Quality Control and Reporting

After data have been entered into the study database, a system of computerized data validation checks will be implemented and applied to the database on a regular basis. *For EDC studies:* Queries are entered, tracked, and resolved through the EDC system directly. *For paper studies:* Query reports (Data Clarification Requests) pertaining to data omissions and discrepancies will be forwarded to the Investigators and study monitors for resolution. The study database will be updated in accordance with the resolved queries. All changes to the study database will be documented.

12.4 Archival of Data

The database is safeguarded against unauthorized access by established security procedures; appropriate backup copies of the database and related software files will be maintained. Databases are backed up by the database administrator in conjunction with any updates or changes to the database.

At critical junctures of the protocol (e.g., production of interim reports and final reports), data for analysis is locked and cleaned per established procedures.

12.4.1 Availability and Retention of Investigational Records

The Investigator must make study data accessible to the monitor, other authorized representatives of the Sponsor (or designee), IRB/IEC, and Regulatory Agency (e.g., FDA) inspectors upon request. A file for each subject must be maintained that includes the signed Informed Consent, HIPAA Authorization and Assent Form and copies of all source documentation related to that subject. The Investigator must ensure the reliability and availability of source documents from which the information on the CRF was derived.

All study documents (patient files, signed informed consent forms, copies of CRFs, Study File Notebook, etc.) must be kept secured for a period of two years following marketing of the investigational product or for two years after centers have been notified that the IND has been discontinued. There may be other circumstances for which the investigator is required to maintain study records and, therefore, the investigator should be contacted prior to removing study records for any reason.

12.4.2 Monitoring

The UCLA Jonsson Comprehensive Cancer Center (JCCC) Data and Safety Monitoring Committee (DSMC) is responsible for monitoring data quality and subject safety for all JCCC institutional clinical studies. A summary of DSMC activities for this study include:

- Review of subject data
- Review of suspected adverse reactions considered “serious”
- Monitoring every six months (depending on study accrual)
- Minimum of a yearly regulatory audit

Monitoring and Reporting Guidelines

Investigators will conduct continuous review of data and subject safety and discuss each subject’s treatment at monthly Site Committee meetings. These discussions are documented in the Site Committee meeting minutes. The discussion will include the number of subjects, significant toxicities in accordance with the protocol, and observed responses.

All institutional Phase 2 or 3 studies are designated with a moderate risk assessment. The data is monitored twice per year with twenty percent of the subjects monitored (or at least three subjects if the calculated value is less than three).

Adverse Event Review and Monitoring
All grade(s) 3-5 adverse events, whether or not unexpected, and whether or not considered to be associated with the use of the study drug, will be entered into OnCore®, UCLA's Clinical Trial Management System.

All grade(s) 3-5 adverse events entered into OnCore® will be reviewed on a monthly basis at the Site Committee meetings. The Site Committee will review and discuss the selected toxicity, the toxicity grade, and the attribution of relationship of the adverse event to the administration of the study drug(s).

In addition, all suspected adverse reactions considered "serious" entered into OnCore®, will be reviewed and monitored by the Data and Safety Monitoring Committee on an ongoing basis and discussed at DSMC meetings, which take place every six weeks.

If a death occurs during the treatment phase of the study or within 30 days after the last administration of the study drug(s) and it is determined to be related either to the study drug(s) or to a study procedure, the Investigator or his/her designee must notify the DSMC Chair within **1 business day** of knowledge of this event. The contact may be by phone or e-mail.

Increase in Adverse Event Rates
If an increase in the frequency of Grade 3 or 4 adverse events (above the rate reported in the Investigator Brochure or package insert) is noted in the study, a report should be submitted to the DSMC at the time the increased rate is identified. The report will indicate if the incidence of adverse events observed in the study is above the range stated in the Investigator Brochure or package insert.

If at any time the Investigator stops enrollment or stops the study due to safety issues, the DSMC Chair and DSMC Manager must be notified within 1 business day via e-mail. The DSMC must receive a formal letter within 10 business days and the CHR must be notified.

13 ADMINISTRATIVE, ETHICAL, REGULATORY CONSIDERATIONS

The study will be conducted according to the Declaration of Helsinki, Protection of Human Volunteers (21 CFR 50), Institutional Review Boards (21 CFR 56), and Obligations of Clinical Investigators (21 CFR 312).

To maintain confidentiality, all laboratory specimens, evaluation forms, reports and other records will be identified by a coded number and initials only. All study records will be kept in a locked file cabinet and code sheets linking a patient's name to a patient identification number will be stored separately in another locked file cabinet. Clinical information will not be released without written permission of the subject, except as necessary for monitoring by the FDA. The Investigator must also comply with all applicable privacy regulations (e.g., Health Insurance Portability and Accountability Act of 1996, EU Data Protection Directive 95/46/EC).

13.1 Protocol Amendments

Any amendment to the protocol will be written by the investigator. Protocol amendments cannot be implemented without prior written IRB/IEC approval except as necessary to eliminate

immediate safety hazards to patients. A protocol amendment intended to eliminate an apparent immediate hazard to patients may be implemented immediately, provided the IRBs are notified within five working days.

13.2 Institutional Review Boards and Independent Ethics Committees

The protocol and consent form will be reviewed and approved by the IRB/IEC of each participating center and BMS prior to study initiation. Serious adverse experiences regardless of causality will be reported to the IRB/IEC in accordance with the standard operating procedures and policies of the IRB/IEC, and the Investigator will keep the IRB/IEC informed as to the progress of the study. The Investigator will obtain assurance of IRB/IEC compliance with regulations.

Any documents that the IRB/IEC may need to fulfill its responsibilities (such as protocol, protocol amendments, Investigator's Brochure, consent forms, information concerning patient recruitment, payment or compensation procedures, or other pertinent information) will be submitted to the IRB/IEC. The IRB/IECs written unconditional approval of the study protocol and the informed consent form will be in the possession of the Investigator before the study is initiated. The IRB/IECs unconditional approval statement will be transmitted by the Investigator to BMS prior to the shipment of study supplies to the site. This approval must refer to the study by exact protocol title and number and should identify the documents reviewed and the date of review.

Protocol and/or informed consent modifications or changes may not be initiated without prior written IRB/IEC approval except when necessary to eliminate immediate hazards to the patients or when the change(s) involves only logistical or administrative aspects of the study. Such modifications will be submitted to the IRB/IEC and written verification that the modification was submitted and subsequently approved should be obtained.

The IRB/IEC must be informed of revisions to other documents originally submitted for review; serious and/or unexpected adverse experiences occurring during the study in accordance with the standard operating procedures and policies of the IRB; new information that may affect adversely the safety of the patients of the conduct of the study; an annual update and/or request for re-approval; and when the study has been completed.

13.3 Informed Consent Form

Informed consent will be obtained in accordance with the Declaration of Helsinki, ICH GCP, US Code of Federal Regulations for Protection of Human Subjects (21 CFR 50.25[a,b], CFR 50.27, and CFR Part 56, Subpart A), the Health Insurance Portability and Accountability Act (HIPAA, if applicable), and local regulations.

The Investigator will prepare the informed consent form, assent and HIPAA authorization prior to submission to the IRB/IEC. The consent form generated by the Investigator must be acceptable to the Sponsor and be approved by the IRB/IEC. The written consent document will embody the elements of informed consent as described in the International Conference on Harmonisation and will also comply with local regulations. The Investigator will send an IRB/IEC-approved copy of the Informed Consent Form to the Sponsor (or designee) for the study file.

A properly executed, written, informed consent will be obtained from each subject prior to entering the subject into the trial. Information should be given in both oral and written form and subjects (or their legal representatives) must be given ample opportunity to inquire about details of the study. If appropriate and required by the local IRB/IEC, assent from the subject will also be obtained. If a subject is unable to sign the informed consent form (ICF) and the HIPAA authorization, a legal representative may sign for the subject. A copy of the signed consent form (and assent) will be given to the subject or legal representative of the subject and the original will be maintained with the subject's records.

The following (re: Post-Study Access) language should be included in your Protocol. It is recommended that as the Sponsor, you also ensure appropriate wording is placed within the Patient Informed Consent:

- *At the end of the study period, Bristol-Myers Squibb Company will not continue to supply study drug to subjects/investigators unless the Sponsor-Investigator chooses to extend their study. The investigator is responsible to ensure that the subject receives appropriate standard of care or other appropriate treatment in the independent medical judgement of the Investigator to treat the condition under study.*

INFORMED CONSENT PROCEDURES

Before protocol-specified procedures are carried out, consenting professionals will explain full details of the protocol and study procedures as well as the risks involved to participants prior to their inclusion in the study. Participants will also be informed that they are free to withdraw from the study at any time. All participants must sign an IRB/PB-approved consent form indicating their consent to participate. This consent form meets the requirements of the Code of Federal Regulations and the Institutional Review Board/Privacy Board of this Center. The consent form will include the following:

1. The nature and objectives, potential risks and benefits of the intended study.
2. The length of study and the likely follow-up required.
3. Alternatives to the proposed study. (This will include available standard and investigational therapies. In addition, patients will be offered an option of supportive care for therapeutic studies.)
4. The name of the investigator(s) responsible for the protocol.
5. The right of the participant to accept or refuse study interventions/interactions and to withdraw from participation at any time.

Before any protocol-specific procedures can be carried out, the consenting professional will fully explain the aspects of patient privacy concerning research specific information. In addition to signing the IRB Informed Consent, all patients must agree to the Research Authorization component of the informed consent form.

Each participant and consenting professional will sign the consent form. The participant must receive a copy of the signed informed consent form.

13.4 Publications

The preparation and submittal for publication of manuscripts containing the study results shall be in accordance with a process determined by mutual written agreement among the BMS and participating institutions. The publication or presentation of any study results shall comply with all applicable privacy laws, including, but not limited to, the Health Insurance Portability and Accountability Act of 1996.

13.5 Investigator Responsibilities

By signing the Agreement of Investigator form, the Investigator agrees to:

1. Conduct the study in accordance with the protocol and only make changes after notifying the Sponsor (or designee), except when to protect the safety, rights or welfare of subjects.
2. Personally conduct or supervise the study (or investigation).
3. Ensure that the requirements relating to obtaining informed consent and IRB review and approval meet federal guidelines, as stated in § 21 CFR, parts 50 and 56.
4. Report to the Sponsor or designee any AEs that occur in the course of the study, in accordance with §21 CFR 312.64.
5. Ensure that all associates, colleagues and employees assisting in the conduct of the study are informed about their obligations in meeting the above commitments.
6. Maintain adequate and accurate records in accordance with §21 CFR 312.62 and to make those records available for inspection with the Sponsor (or designee).
7. Ensure that an IRB that complies with the requirements of §21 CFR part 56 will be responsible for initial and continuing review and approval of the clinical study.
8. Promptly report to the IRB and BMS (or designee) all changes in the research activity and all unanticipated problems involving risks to subjects or others (to include amendments and IND safety reports).
9. Seek IRB approval before any changes are made in the research study, except when necessary to eliminate hazards to the patients/subjects.
10. Comply with all other requirements regarding the obligations of clinical investigators and all other pertinent requirements listed in § 21 CFR part 312.

14 References

1. Hidalgo, M., *Pancreatic cancer.* N Engl J Med, 2010. **362**(17): p. 1605-17.
2. Varadhachary, G.R., et al., *Borderline resectable pancreatic cancer: definitions, management, and role of preoperative therapy.* Ann Surg Oncol, 2006. **13**(8): p. 1035-46.
3. Kim, S.S., et al., *Preoperative FOLFIRINOX for borderline resectable pancreatic cancer: Is radiation necessary in the modern era of chemotherapy?* J Surg Oncol, 2016. **114**(5): p. 587-596.
4. Kleeff, J., et al., *Pancreatic cancer microenvironment.* Int J Cancer, 2007. **121**(4): p. 699-705.
5. Feig, C., et al., *The pancreas cancer microenvironment.* Clin Cancer Res, 2012. **18**(16): p. 4266-76.
6. Joyce, J.A. and J.W. Pollard, *Microenvironmental regulation of metastasis.* Nat Rev Cancer, 2009. **9**(4): p. 239-52.
7. Neesse, A., et al., *Stromal biology and therapy in pancreatic cancer.* Gut, 2011. **60**(6): p. 861-8.
8. Chu, G.C., et al., *Stromal biology of pancreatic cancer.* J Cell Biochem, 2007. **101**(4): p. 887-907.
9. Wehr, A.Y., et al., *Analysis of the human pancreatic stellate cell secreted proteome.* Pancreas, 2011. **40**(4): p. 557-66.
10. Haber, P.S., et al., *Activation of pancreatic stellate cells in human and experimental pancreatic fibrosis.* Am J Pathol, 1999. **155**(4): p. 1087-95.
11. Phillips, P., *Pancreatic stellate cells and fibrosis*, in *Pancreatic Cancer and Tumor Microenvironment*, P.J. Grippo and H.G. Munshi, Editors. 2012: Trivandrum (India).
12. Hwang, R.F., et al., *Cancer-associated stromal fibroblasts promote pancreatic tumor progression.* Cancer Res, 2008. **68**(3): p. 918-26.
13. Murphy JE, Wo JY, Ryan DP, Jiang W, Yeap BY, Drapek LC, Blaszkowsky LS, Kwak EL, Allen JN, Clark JW, Faris JE, Zhu AX, Goyal L, Lillemoe KD, DeLaney TF, Fernández-Del Castillo C, Ferrone CR, Hong TS. *Total Neoadjuvant Therapy With FOLFIRINOX Followed by Individualized Chemoradiotherapy for Borderline Resectable Pancreatic Adenocarcinoma: A Phase 2 Clinical Trial*. JAMA Oncol. 2018 May 3. doi: 10.1001/jamaoncol.2018.0329.
14. Ho, C.K., et al., *Complications of pancreatic surgery.* HPB (Oxford), 2005. **7**(2): p. 99-108.
15. Lin, J.W., et al., *Risk factors and outcomes in postpancreaticoduodenectomy pancreaticocutaneous fistula.* J Gastrointest Surg, 2004. **8**(8): p. 951-9.
16. Bartoli, F.G., et al., *Pancreatic fistula and relative mortality in malignant disease after pancreaticoduodenectomy. Review and statistical meta-analysis regarding 15 years of literature.* Anticancer Res, 1991. **11**(5): p. 1831-48.
17. Bassi, C., et al., *Management of complications after pancreaticoduodenectomy in a high volume centre: results on 150 consecutive patients.* Dig Surg, 2001. **18**(6): p. 453-7; discussion 458.
18. Poon, R.T., et al., *Prevention of pancreatic anastomotic leakage after pancreaticoduodenectomy.* Am J Surg, 2002. **183**(1): p. 42-52.
19. Tsiotos, G.G., M.B. Farnell, and M.G. Sarr, *Are the results of pancreatectomy for pancreatic cancer improving?* World J Surg, 1999. **23**(9): p. 913-9.

20. Balcom, J.H.t., et al., *Ten-year experience with 733 pancreatic resections: changing indications, older patients, and decreasing length of hospitalization.* Arch Surg, 2001. **136**(4): p. 391-8.
21. Yeo, C.J., et al., *Six hundred fifty consecutive pancreaticoduodenectomies in the 1990s: pathology, complications, and outcomes.* Ann Surg, 1997. **226**(3): p. 248-57; discussion 257-60.
22. Bassi, C., et al., *Postoperative pancreatic fistula: an international study group (ISGPF) definition.* Surgery, 2005. **138**(1): p. 8-13.
23. Callery, M.P., et al., *A prospectively validated clinical risk score accurately predicts pancreatic fistula after pancreatoduodenectomy.* J Am Coll Surg, 2013. **216**(1): p. 1-14.
24. Pratt, W.B., et al., *Clinical and economic validation of the International Study Group of Pancreatic Fistula (ISGPF) classification scheme.* Ann Surg, 2007. **245**(3): p. 443-51.
25. Conroy, T., et al., *FOLFIRINOX versus gemcitabine for metastatic pancreatic cancer.* N Engl J Med, 2011. **364**(19): p. 1817-25.
14. Erkan, M., et al., *Cancer-stellate cell interactions perpetuate the hypoxia-fibrosis cycle in pancreatic ductal adenocarcinoma.* Neoplasia, 2009. **11**(5): p. 497-508.
15. Erkan, M., et al., *Tumor microenvironment and progression of pancreatic cancer.* Exp Oncol, 2010. **32**(3): p. 128-31.
16. Erkan, M., et al., *The activated stroma index is a novel and independent prognostic marker in pancreatic ductal adenocarcinoma.* Clin Gastroenterol Hepatol, 2008. **6**(10): p. 1155-61.
17. Jenkins, R.H., et al., *Myofibroblastic differentiation leads to hyaluronan accumulation through reduced hyaluronan turnover.* J Biol Chem, 2004. **279**(40): p. 41453-60.
18. Provenzano, P.P., et al., *Enzymatic targeting of the stroma ablates physical barriers to treatment of pancreatic ductal adenocarcinoma.* Cancer Cell, 2012. **21**(3): p. 418-29.
19. Von Hoff, D.D., et al., *Gemcitabine plus nab-paclitaxel is an active regimen in patients with advanced pancreatic cancer: a phase I/II trial.* J Clin Oncol, 2011. **29**(34): p. 4548-54.
20. Desai, N., et al., *Increased antitumor activity, intratumor paclitaxel concentrations, and endothelial cell transport of cremophor-free, albumin-bound paclitaxel, ABI-007, compared with cremophor-based paclitaxel.* Clin Cancer Res, 2006. **12**(4): p. 1317-24.
21. Shepard, H.M.F., G. I.; Rybak, M. E.; Ramanathan, R. K.; Von Hoff, D. D. ; Infante, J.R.; Rosen, L. S. Leff, J. , *Targeting hyaluronan (HA) in tumor stroma: Translational evaluation of pegylated hyaluronidase (PEGPH20, P) in animal models and patients (PTS) with advanced solid tumors.*, in *2010 ASCO-NCI-EORTC Annual Meeting on Molecular Markers in Cancer*. 2010 Florida.
22. Hingorani, S.R.H., W.P.; Beck, J.T.; Berdov, B.A.; Wagner, S.A.; Pshevlotsky, E.M.; Tiulandin, S.; Gladkov, O.; Holcombe, R.F.; Jiang, P.; Maneval, D.C.; Zhu, J. Devoe, C.E., *A phase Ib study of gemcitabine plus PEGPH20 (pegylated recombinant human hyaluronidase) in patients with stage IV previously untreated pancreatic cancer.* J Clin Oncol 2013. **31**(suppl; abstr 4010).
23. Olive, K.P., et al., *Inhibition of Hedgehog signaling enhances delivery of chemotherapy in a mouse model of pancreatic cancer.* Science, 2009. **324**(5933): p. 1457-61.
24. Frese, K.K., et al., *nab-Paclitaxel potentiates gemcitabine activity by reducing cytidine deaminase levels in a mouse model of pancreatic cancer.* Cancer Discov, 2012. **2**(3): p. 260-9.

25. Von Hoff, D.D., et al., *Increased survival in pancreatic cancer with nab-paclitaxel plus gemcitabine.* N Engl J Med, 2013. **369**(18): p. 1691-703.
26. Conroy T et al. *PRODIGE 24/CCTG PA.6, an Unicancer GI trial: a multicenter international randomized phase III trial of adjuvant mFOLFIRINOX versus gemcitabine (gem) in patients with resected pancreatic ductal adenocarcinomas.* Proc ASCO 2018.
27. Sharpe AH, Wherry EJ, Ahmed R, et al. *The function of programmed cell death 1 and its ligands in regulating autoimmunity and infection.* Nature Immunol. 2007;8:237-45.
28. Pardoll DM. T*he blockade of immune checkpoints in cancer immunotherapy*. Nature 2012;12:252-64.
