# Supplementary material for: Neoadjuvant modified FOLFIRINOX plus nivolumab in borderline-resectable pancreatic ductal adenocarcinoma: a pilot phase 1 trial
Source: Nat Commun. 2026 Jan 31;17:2232. doi: 10.1038/s41467-026-68976-2 (PMC12963377; doi:10.1038/s41467-026-68976-2)
Supplement: Supplementary file 2 — Description of Additional Supplementary Files [file 41467_2026_68976_MOESM2_ESM.pdf]

Description of Additional Supplementary Files

Supplementary Data 1: Clinical Information

Supplementary Data 2: Immunhistochemistry Data - All Aggregates

Supplementary Data 3: Immunohistochemistry Data - Patient Level

Supplementary Data 4: Spatial Transcriptomics Data
