# Supplementary material for: Neoadjuvant modified FOLFIRINOX plus nivolumab in borderline-resectable pancreatic ductal adenocarcinoma: a pilot phase 1 trial
Source: Nat Commun. 2026 Jan 31;17:2232. doi: 10.1038/s41467-026-68976-2 (PMC12963377; doi:10.1038/s41467-026-68976-2)
Supplement: Supplementary file 4 — Reporting Summary [file 41467_2026_68976_MOESM4_ESM.pdf]

nature portfolio

Corresponding author(s): Timothy Donahue

Last updated by author(s): Dec 15, 2025

Reporting Summary

Nature Portfolio wishes to improve the reproducibility of the work that we publish. This form provides structure for consistency and transparency in reporting. For further information on Nature Portfolio policies, see our Editorial Policies and the Editorial Policy Checklist.

Statistics

For all statistical analyses, confirm that the following items are present in the figure legend, table legend, main text, or Methods section.

n/a	Confirmed	
☐	☒	The exact sample size (n) for each experimental group/condition, given as a discrete number and unit of measurement
☐	☒	A statement on whether measurements were taken from distinct samples or whether the same sample was measured repeatedly
☐	☒	The statistical test(s) used AND whether they are one- or two-sided<br>*Only common tests should be described solely by name; describe more complex techniques in the Methods section.*
☐	☒	A description of all covariates tested
☐	☒	A description of any assumptions or corrections, such as tests of normality and adjustment for multiple comparisons
☐	☒	A full description of the statistical parameters including central tendency (e.g. means) or other basic estimates (e.g. regression coefficient) AND variation (e.g. standard deviation) or associated estimates of uncertainty (e.g. confidence intervals)
☐	☒	For null hypothesis testing, the test statistic (e.g. F, t, r) with confidence intervals, effect sizes, degrees of freedom and P value noted<br>*Give P values as exact values whenever suitable.*
☒	☐	For Bayesian analysis, information on the choice of priors and Markov chain Monte Carlo settings
☒	☐	For hierarchical and complex designs, identification of the appropriate level for tests and full reporting of outcomes
☒	☐	Estimates of effect sizes (e.g. Cohen's d, Pearson's r), indicating how they were calculated

Our web collection on statistics for biologists contains articles on many of the points above.

Software and code

Policy information about availability of computer code

Data collection	Clinical data were collected directly from electronic medical records and no code or software was used.
Data analysis	No custom software or code were developed for this manuscript. All software used for analyses have been reported in the Methods

For manuscripts utilizing custom algorithms or software that are central to the research but not yet described in published literature, software must be made available to editors and reviewers. We strongly encourage code deposition in a community repository (e.g. GitHub). See the Nature Portfolio guidelines for submitting code & software for further information.

Data

Policy information about availability of data

All manuscripts must include a data availability statement. This statement should provide the following information, where applicable:

- Accession codes, unique identifiers, or web links for publicly available datasets
- A description of any restrictions on data availability
- For clinical datasets or third party data, please ensure that the statement adheres to our policy

Data supporting the findings of this study are available in the article, the supplementary materials, or the source data provided with this paper. The RNA-seq data generated in this study have been deposited in the Gene Expression Omnibus under accession number GSE313101. The spatial transcriptomics data generated in this study have been deposited in the Gene Expression Omnibus under accession number GSE313662. A redacted study protocol is provided in the Supplementary Information.

Research involving human participants, their data, or biological material

Policy information about studies with human participants or human data. See also policy information about sex, gender (identity/presentation), and sexual orientation and race, ethnicity and racism.

Reporting on sex and gender	Sex (biological attribute) was recorded for all participants, and only male and female categories were reported. All patients were adults with a confirmed diagnosis of borderline-resectable pancreatic ductal adenocarcinoma (PDAC). Sex-based analyses were not performed due to limited sample size and because sex was not hypothesized to impact the primary or secondary endpoints. Overall numbers by sex can be found in Table 1 of the manuscript. Gender identity was not collected.
Reporting on race, ethnicity, or other socially relevant groupings	Race and ethnicity were not recorded as part of the clinical trial and thus not included in any analyses. The study focused on biologic and clinical endpoints, including safety, progression-free survival, and pathologic response. Consequently, race and ethnicity were not used to define subgroups or interpret findings.
Population characteristics	The study population consisted of adults with borderline resectable pancreatic cancer, confirmed by cytology or histology and reviewed by a multidisciplinary tumor board. Eligibility criteria included ECOG performance status 0–1 and adequate organ function. Full inclusion and exclusion criteria are detailed in the protocol (Redacted Trial Protocol, Sections 6.1 and 6.2). Patient demographics including age and performance status are provided in Table 1 of the manuscript. No pediatric patients or individuals from vulnerable populations were included.
Recruitment	Participants were recruited from a single academic center (UCLA Jonsson Comprehensive Cancer Center). Patients were approached based on diagnosis and tumor board eligibility review. All patients provided written informed consent. There is potential for self-selection bias due to the single-site design and the nature of clinical trial participation, but all eligible patients were offered participation. Details on recruitment are described in the Methods section of the manuscript (lines 95–117) and protocol.
Ethics oversight	The study was approved by the UCLA Institutional Review Board and conducted in accordance with ICH-GCP and the Declaration of Helsinki. Oversight was also provided by the UCLA Data Safety Monitoring Board (DSMB). Ethics approval is stated explicitly in the manuscript Methods section (lines 88–93) and on the trial protocol signature page.

Note that full information on the approval of the study protocol must also be provided in the manuscript.

Field-specific reporting

Please select the one below that is the best fit for your research. If you are not sure, read the appropriate sections before making your selection.

[x] Life sciences [ ] Behavioural & social sciences [ ] Ecological, evolutionary & environmental sciences

For a reference copy of the document with all sections, see nature.com/documents/nr-reporting-summary-flat.pdf

Life sciences study design

All studies must disclose on these points even when the disclosure is negative.

Sample size	All available samples from the clinical trial were used for bulk RNA-seq and immunohistochemistry analyses. For spatial transcriptomics, six post-treatment samples were selected based on tissue quality and representative histopathology. Samples with complete responses or basal-like transcriptional profiles were excluded to avoid confounding interpretation of lymphoid aggregate architecture. An additional three samples were profiled using an earlier spatial panel with a reduced gene set and were not combined with the 5,101-gene panel sample analysis due to incompatibility.
Data exclusions	No data were excluded from the primary clinical analyses. For spatial transcriptomics, only samples with sufficient viable tumor tissue and high-quality FFPE preservation were included. Predefined thresholds for tissue quality and RNA integrity were applied before inclusion. Cells with fewer than 10 detected genes or fewer than 20 transcripts were excluded from downstream spatial analyses as described in the Methods. All computations were performed with R (version 2025) or Microsoft Excel (version 16) and all statistical tests were performed using GraphPad Prism (version 10).
Replication	RNA-seq and IHC experiments were successfully performed on all eligible samples, including matched pre- and post-treatment specimens where available. Replication was confirmed by consistent findings across independent samples and analytical platforms. For spatial transcriptomics, reproducibility was ensured through consistent detection of cluster-specific markers across samples and validation by a board-certified pathologist using matched H&E images.
Randomization	This was a single-arm, open-label clinical trial without random assignment to treatment groups. All enrolled patients received the same intervention (mFOLFIRINOX + nivolumab). Comparison groups (e.g., mFOLFIRINOX-only historical controls) were selected retrospectively based on disease stage and treatment timing to control for key covariates.
Blinding	Blinding was not possible during patient treatment or clinical outcome collection due to the single-arm nature of the study.

Reporting for specific materials, systems and methods

We require information from authors about some types of materials, experimental systems and methods used in many studies. Here, indicate whether each material, system or method listed is relevant to your study. If you are not sure if a list item applies to your research, read the appropriate section before selecting a response.

Materials & experimental systems

n/a	Involved in the study
☐	☒ Antibodies
☒	☐ Eukaryotic cell lines
☒	☐ Palaeontology and archaeology
☒	☐ Animals and other organisms
☐	☒ Clinical data
☒	☐ Dual use research of concern
☒	☐ Plants

Methods

n/a	Involved in the study
☒	☐ ChIP-seq
☒	☐ Flow cytometry
☒	☐ MRI-based neuroimaging

Antibodies

Antibodies used	All antibodies and manufacturers are described in a Key Resources table at the end of the Supplementary Information file
Validation	All primary antibodies were validated by the University of California, Los Angeles Translational Pathology Core Laboratory. Staining patterns were evaluated by a board-certified pathologist and were consistent with the intended antibody targets

Clinical data

Policy information about clinical studies

All manuscripts should comply with the ICMJE guidelines for publication of clinical research and a completed CONSORT checklist must be included with all submissions.

Clinical trial registration	NCT03970252
Study protocol	The full trial protocol is provided as a supplementary file with the manuscript and available upon request. It was approved by the UCLA Institutional Review Board and registered with ClinicalTrials.gov.
Data collection	Data were collected at the University of California, Los Angeles (UCLA) Jonsson Comprehensive Cancer Center between March 2022 and December 2024. Clinical, laboratory, and imaging data were obtained prospectively at baseline and regular follow-up visits. Biospecimens were collected from pretreatment biopsies and surgical resection specimens and processed for molecular and histological analyses as outlined in the Methods.
Outcomes	Primary outcomes were prospectively defined as treatment safety (grade ≥3 adverse events) and feasibility (rate of surgical resection following therapy). Secondary outcomes included progression-free survival (PFS), overall survival (OS), radiographic and biochemical response rates (RECIST v1.1, CA19-9), and pathologic response (CAP score). Outcomes were assessed as per protocol and detailed in the manuscript Methods section.

Plants

Seed stocks	*Report on the source of all seed stocks or other plant material used. If applicable, state the seed stock centre and catalogue number. If plant specimens were collected from the field, describe the collection location, date and sampling procedures.*
Novel plant genotypes	*Describe the methods by which all novel plant genotypes were produced. This includes those generated by transgenic approaches, gene editing, chemical/radiation-based mutagenesis and hybridization. For transgenic lines, describe the transformation method, the number of independent lines analyzed and the generation upon which experiments were performed. For gene-edited lines, describe the editor used, the endogenous sequence targeted for editing, the targeting guide RNA sequence (if applicable) and how the editor was applied.*
Authentication	*Describe any authentication procedures for each seed stock used or novel genotype generated. Describe any experiments used to assess the effect of a mutation and, where applicable, how potential secondary effects (e.g. second site T-DNA insertions, mosiacism, off-target gene editing) were examined.*
